# Flexible Crystal Heterojunctions of Low-Dimensional Organic Metal Halides Enabling Color-Tunable Space-Resolved Optical Waveguides

**DOI:** 10.34133/research.0259

**Published:** 2023-10-30

**Authors:** Yuhang Lin, Shuya Liu, Dongpeng Yan

**Affiliations:** Beijing Key Laboratory of Energy Conversion and Storage Materials, and Key Laboratory of Radiopharmaceuticals, Ministry of Education, College of Chemistry, Beijing Normal University, Beijing 100875, China.

## Abstract

Molecular luminescent materials with optical waveguide have wide application prospects in light-emitting diodes, sensors, and logic gates. However, the majority of traditional optical waveguide systems are based on brittle molecular crystals, which limited the fabrication, transportation, storage, and adaptation of flexible devices under diverse application situations. To date, the design and synthesis of photofunctional materials with high flexibility, novel optical waveguide, and multi-port color-tunable emission in the same solid-state system remain an open challenge. Here, we have constructed new types of zero-dimensional organic metal halides (Au-4-dimethylaminopyridine [DMAP] and In-DMAP) with a rarely high elasticity and rather low loss coefficients for optical waveguide. Theoretical calculations on the intermolecular interactions showed that the high elasticity of 2 molecular crystalline materials was original from their herringbone structure and slip plane. Based on one-dimensional flexible microrods of 2 crystals and the 2-dimensional microplate of the Mn-DMAP, heterojunctions with multi-color and space-resolved optical waveguides have been fabricated. The formation mechanism of heterojunctions is based on the surface selective growth on account of the low lattice mismatch ratio between contacting crystal planes. Therefore, this work describes the first attempt to the design of metal-halide-based crystal heterojunctions with high flexibility and optical waveguide, expanding the prospects of traditional luminescent materials for smart optical devices, such as logic gates and multiplexers.

## Introduction

Molecular luminescent materials have attracted widespread attention due to their applications in light-emitting diodes [1,2], molecular imaging [3–5], sensors [6–8], actuators [9,10], anti-counterfeiting [11,12], and photodynamic therapy [13,14]. As burgeoning luminescent systems, low-dimensional organic metal halides (OMHs) have been demonstrated with great potentials in solar cells [15,16], light-emitting diodes [17–20], and information encryption [21,22], among many others. For the information communications, low-dimensional OMHs have shown excellent optical waveguide performance due to their highly ordered structures that can reduce the optical loss during propagation [23–25], and are considered as an important direction for the high-performance information safety [26,27] and optical storage [28,29]. Moreover, the wide selection of inorganic metal halides and organic cations provides high tunability of both crystal structures and electron energy levels in OMHs [30,31]. Multiple intermolecular interactions between different components (such as electrostatic interactions, hydrogen bonds, and π–π stacking) ensure their high stability [32–34]. However, the majority of current research on low-dimensional OMHs is restricted by brittle molecular crystals [24,26,30,35–38], which have substantial limitations in fabrication, transportation, storage, and adaptation of device under complex situations, that hamper the practical applications. Therefore, the development of highly flexible and robust low-dimensional OMHs is necessary toward high-performance optical waveguides and reversible mechanical inductors.

Several flexible molecular materials have been developed in recent years, but research has mainly focused on purely organic single crystals [39–43]. For example, the caffeine co-crystals with elastic bending properties by Ghosh and Reddy [44] are considered as a pioneering report in the research of flexible crystals. Then, Hayashi and Koizumi [45] developed the first π-conjugated molecule 1,4-bis[2-(4-methylthienyl)]-2,3,5,6-tetrafluorobenzene with intense fluorescence emission and elastic properties, and potential applications of such flexible crystal in photoluminescence (PL) were further developed. Following these findings, many flexible organic crystal materials have been reported, among which a series of works by Zhang and co-workers have provided a direction for the combination of flexibility and waveguiding properties [46–48], promoting research on flexible optical waveguides. Although these investigations have shown the potential applications of molecular crystals for optical devices (such as photonic routers, logic gates, modulators, and multiplexers), there is still a need to explore new flexible systems with multifunctional properties to solve problems of single light-emitting color and single port of existing materials. In this context, we expected that the OMH-based heterojunction would serve as an ideal system to achieve multi-port and multi-color flexible optical waveguide performance.

**Fig. 1.**
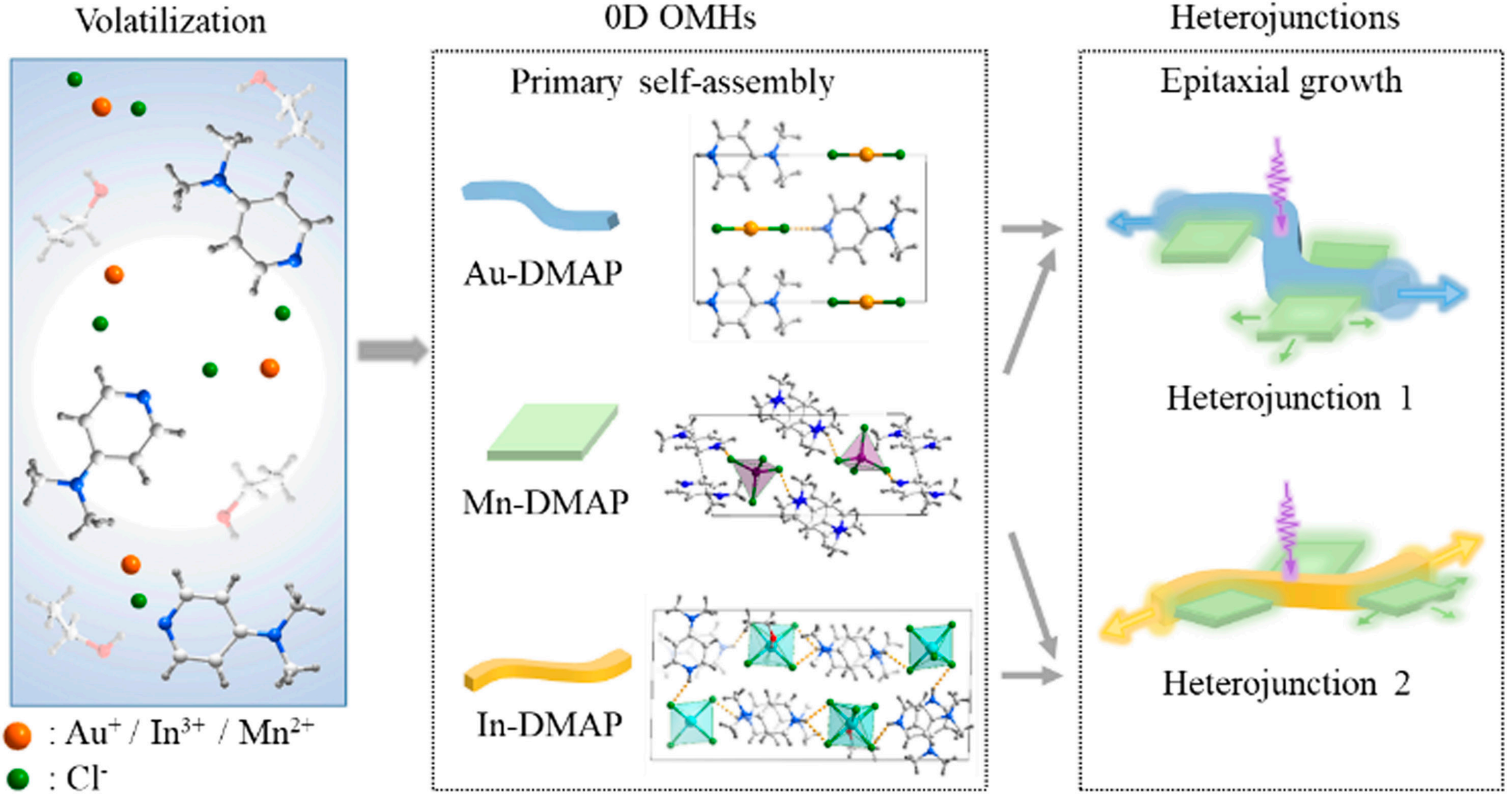
Schemes of formation, crystal stacking pattern, and the demonstration of the optical waveguiding performance of Au-DMAP, Mn-DMAP, In-DMAP, and 2 crystal heterojunctions.

Herein, we chose organic molecule 4-dimethylaminopyridine (DMAP) as a phosphor unit to assemble into 3 zero-dimensional (0D) OMHs (namely, Au-DMAP, Mn-DMAP, and In-DMAP) with metal halides AuCl, InCl_3_, and MnCl_2_ via both hydrothermal and solvent volatilization methods (Fig. 1 and Supplementary Materials). The selection of 3 different metal halide units is based on the expectation that their changeable electronic configurations could result in different degrees of charge transfer (CT) between inorganic and organic components, and thus further lead to different luminescence. Au-DMAP and In-DMAP have excellent one-dimensional (1D) optical waveguide properties and high elasticity that are rather rare in current OMHs. Besides, Mn-DMAP microcrystals have higher luminescence quantum yields with 2-dimensional (2D) optical waveguides. Through an epitaxial-growth method, 2 types of flexible optical waveguide heterojunctions were obtained as Au-DMAP/Mn-DMAP and In-DMAP/Mn-DMAP, respectively (Fig. 1). Both heterojunctions show the advantages of low optical loss coefficient (OLC), high elasticity, and multi-port color-tunable emission, which have great potentials in intelligent optical devices and flexible photonic materials.

## Results

### Construction and mechanical characterization of OMHs

In order to obtain new types of OMHs with high elastic performance, 2 characteristic structures, namely, herringbone [41,49] and slip plane [39,40,50], are generally considered to potentially have flexible and/or elastic ability. New 0D OMHs (Au-DMAP and In-DMAP) with these designed structures were successfully synthesized by the hydrothermal reaction or solvent volatilization, respectively.

**Fig. 2.**
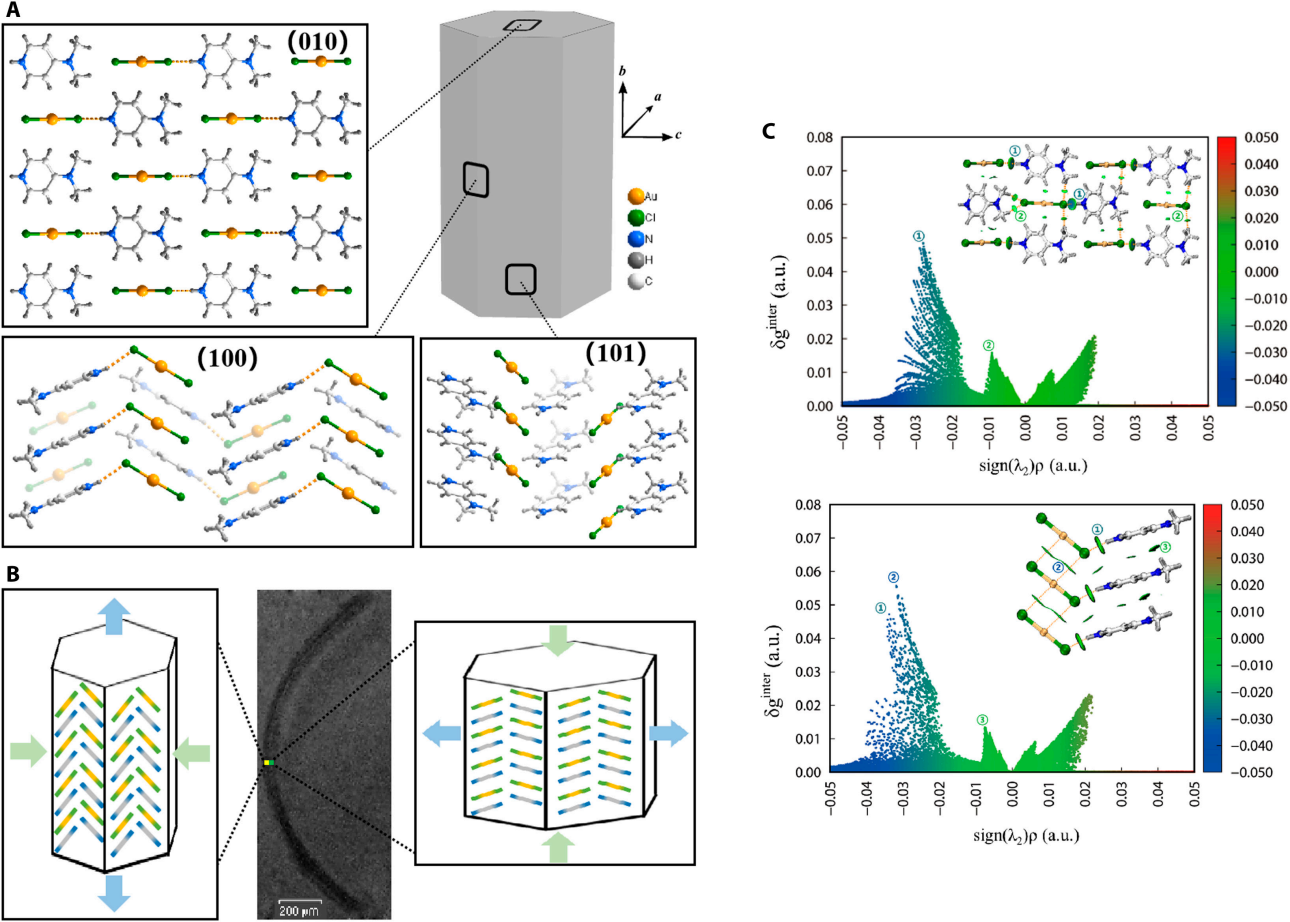
Crystal structure and elastic bending mechanism of Au-DMAP. (A) Packing views of the (100), (010), and (101) planes showing ions stacking and herringbone arrangements, respectively. The molecules shown in gray shade in the packing diagrams are from a column behind. (B) Plausible mechanism of elastic bending involving stretch (left) and compression (right) of outer and inner arcs, respectively, along the long axis of the bent crystal. (C) Independent gradient model based on Hirshfeld partition of Au-DMAP was used to calculate the weak interactions of the (010) plane (left) and the (100) plane (right).

The crystal structures of Au-DMAP and In-DMAP were measured by single-crystal x-ray diffraction (SCXRD, Table S1). Au-DMAP belongs to the orthogonal space group *P*mn2_1_. In the crystal, AuCl_2_^−^ anion and DMAP^+^ cation each stack parallel along the [010] axis or the long axis of the crystal to form a columnar structure, and the dihedral angles of DMAP^+^ and AuCl_2_^−^ with the (010) plane are 23.37° and 32.39°, respectively. The different adjacent stacked columns are tilted with respect to each other to form a herringbone structure, as connected by N-H...Cl hydrogen bonds (2.352 Å, Fig. 2A). Such columnar stacking makes the weak interaction planes of one row to overlap with the next one; thus, the structure lacks a slip-prone plane. Similar to the herringbone structure in elastic organic crystals, Worthy et al. [51] have used a synchrotron diffraction to determine that the elastic bending of such structure occurs *via* the rotation of molecules relative to each other, in which the mechanism can also be applied to Au-DMAP. When Au-DMAP bends under external stress, the outer arc of the crystal is under tensile stress, and the rotation away from the (010) plane of the ions promotes the stretching of the outer structure, while the inner arc is under compressive stress, and the rotation in the opposite direction promotes the compression of the inner arc structure (Fig. 2B). After releasing the external stress, the ions rebound to the direction of the initial structure, helping the crystal to release the elastic deformation. Research has generally suggested that the high elasticity of flexible crystal materials originates from the buffer regions in the crystal structure, formed by weak interactions that accommodate the elastic strain energy [52–54]. To better understand the role of intermolecular interactions on the high elasticity of 0D OMHs, we have quantitatively analyzed the intermolecular forces in the crystal structure using Gaussian 09 software, and built an independent gradient model based on Hirshfeld partition (IGMH) (Fig. 2C). The size of the equivalence surface between the weak interactions is proportional to the magnitude of the weak interactions and is shown quantitatively in the scatter plot. The major weak interaction is the N-H...Cl hydrogen bond, while other interactions within the stacked columns along the long-axis direction are relatively weaker, including molecular stacking and electrostatic interactions between DMAP^+^ and AuCl_2_^−^, which also contribute to the building of buffer regions [49], thereby facilitating the crystal to accommodate elastic deformation.

**Fig. 3.**
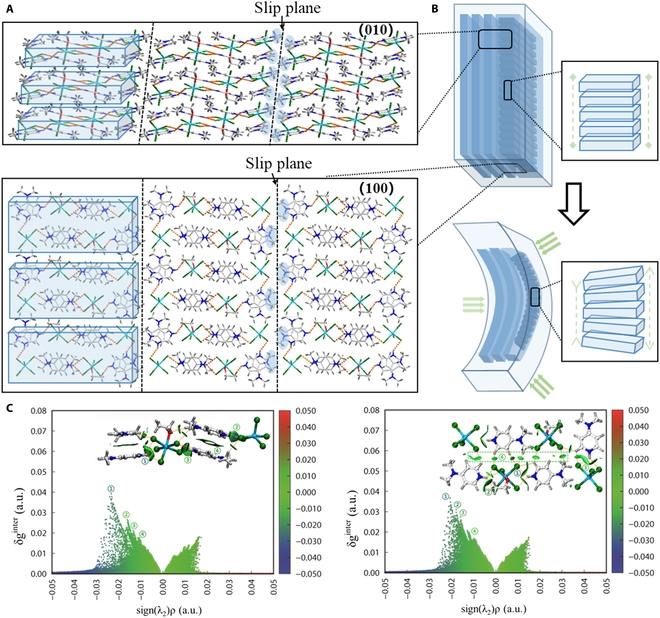
Crystal structure and elastic bending mechanism of In-DMAP. (A) Packing views of the (100) and (010) planes showing ions stacking and slip plane. (B) Illustration of the elastic bending mechanism, including the tilting and orientation adjustment of the ions. (C) Independent gradient model based on Hirshfeld partition of In-DMAP, to evaluate the interactions of the (010) plane (left) and the (100) plane (right).

The space group of In-DMAP is triclinic P with 2 equal proportions of metal halide anions InCl_5_^2−^ and [InCl_5_(EtOH)]^2−^ in the crystal, which are separated by the cationic DMAP^+^ and alternated sequentially in the (100) plane (Fig. 3A). Within the unit cell, the ions are connected by N-H...Cl bonds (2.426 Å to 2.649 Å, Table S2), while the ions between different cells are connected by weaker C-H...Cl bonds and π–π stacking interactions. In addition, the interactions between ions along the (001) plane are substantially weaker than other directions, hence providing a potential slip plane when under external stress (Fig. 3A), while the stronger interactions within the crystal cell ensure the stability of the structure beyond the slip plane, confirming that the In-DMAP has a certain buffering ability. This structure has been found in the majority of plastic and elastic flexible crystals reported up to now [55–58], indicating that the slip planes play a key role in the design of In-DMAP to obtain high elastic performance. The π–π interactions within In-DMAP allow the ions to adapt the crystal deformation due to external stress stimulus by tilting and slight reorientation (Fig. 3B), while the (001) slip plane allows the ions to move along the [100] direction (the long axis of the crystal) during mechanical bending. The same quantitative analysis of the intermolecular interactions in the In-DMAP was performed and an IGMH profile is shown in Fig. 3C. The IGMH analysis exhibits that the N-H...Cl bonding forces in the crystal are substantially stronger than the C-H...Cl bonding and π–π stacking forces, which effectively assist to prove that the high elasticity originated from the (001) slip plane in In-DMAP.

As a comparison, the crystal structure of Mn-DMAP was further detected. Mn-DMAP adopts the triclinic P space group, wherein one MnCl^2−^ tetrahedron anion was surrounded by 4 DMAP^+^ cations, such that the MnCl^2−^ ions are separated from each other (Fig. S2). In contrast to the other 2 crystals, the DMAP^+^ cations in Mn-DMAP are not stacked along one direction, but in 2 orthogonal ways ([100] and [010] directions) to form a column respectively, and the adjacent columns are stacked vertically with each other along the [001] direction (Fig. S2). Such a vertical and tight rigid crystal structure lacks structural buffer regions that accommodate elastic deformation, making the Mn-DMAP crystal easily fracture when stimulated by external stress. However, on the other hand, this tightly stacked rigid structure can effectively suppress the nonradiative relaxation due to the lack of molecular rotation in the excited state, which leads to a high photoluminescence quantum yield (PLQY) for Mn-DMAP.

**Fig. 4.**
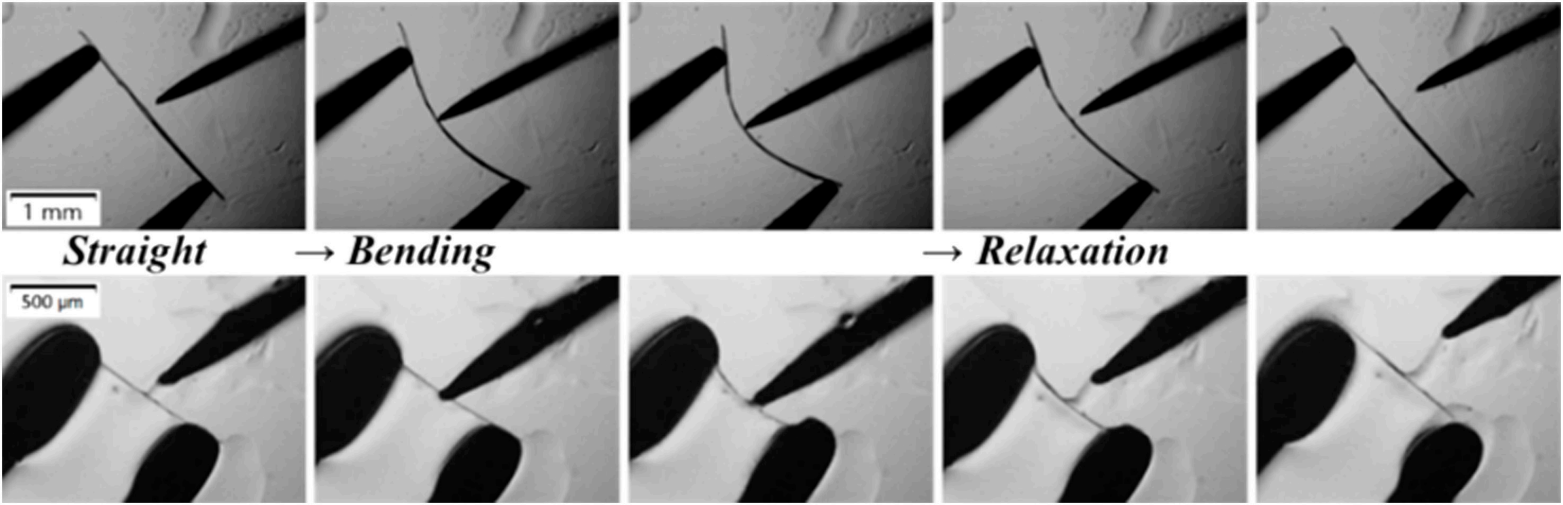
Bending of 2 elastic materials Au-DMAP (top) and In-DMAP (bottom) under external mechanical stress. Scale bars: 1 mm (top) and 500 μm (bottom).

To verify the elastic characteristic of the structural design, a mechanical test was performed on Au-DMAP and In-DMAP under an optical microscope (Fig. 4 and Movies S1 and S2). When Au-DMAP and In-DMAP are under certain external mechanical stress, the straight crystals can be bent without any rupture or fracture, confirming the obviously flexible properties of 2 crystals. After releasing the external stress, the crystals can quickly return to their original straight shape without any permanent damage. This reversible bending-relaxation cycling can be repeated many times, which demonstrates their high elasticity and durability. When the bending reaches the limit, the crystal will fracture. In order to quantify the maximum degree to that when both elastic crystals can be bent, mathematical modeling was used to calculate the limiting curvature at the fracture point. According to the equation (*ε_n_* = i/2*r*) [59], the elastic strain (*ε_n_*) of Au-DMAP and In-DMAP can be calculated (Fig. S1) as 8.02% and 7.71%, respectively.

### Luminescence and optical waveguide properties

**Fig. 5.**
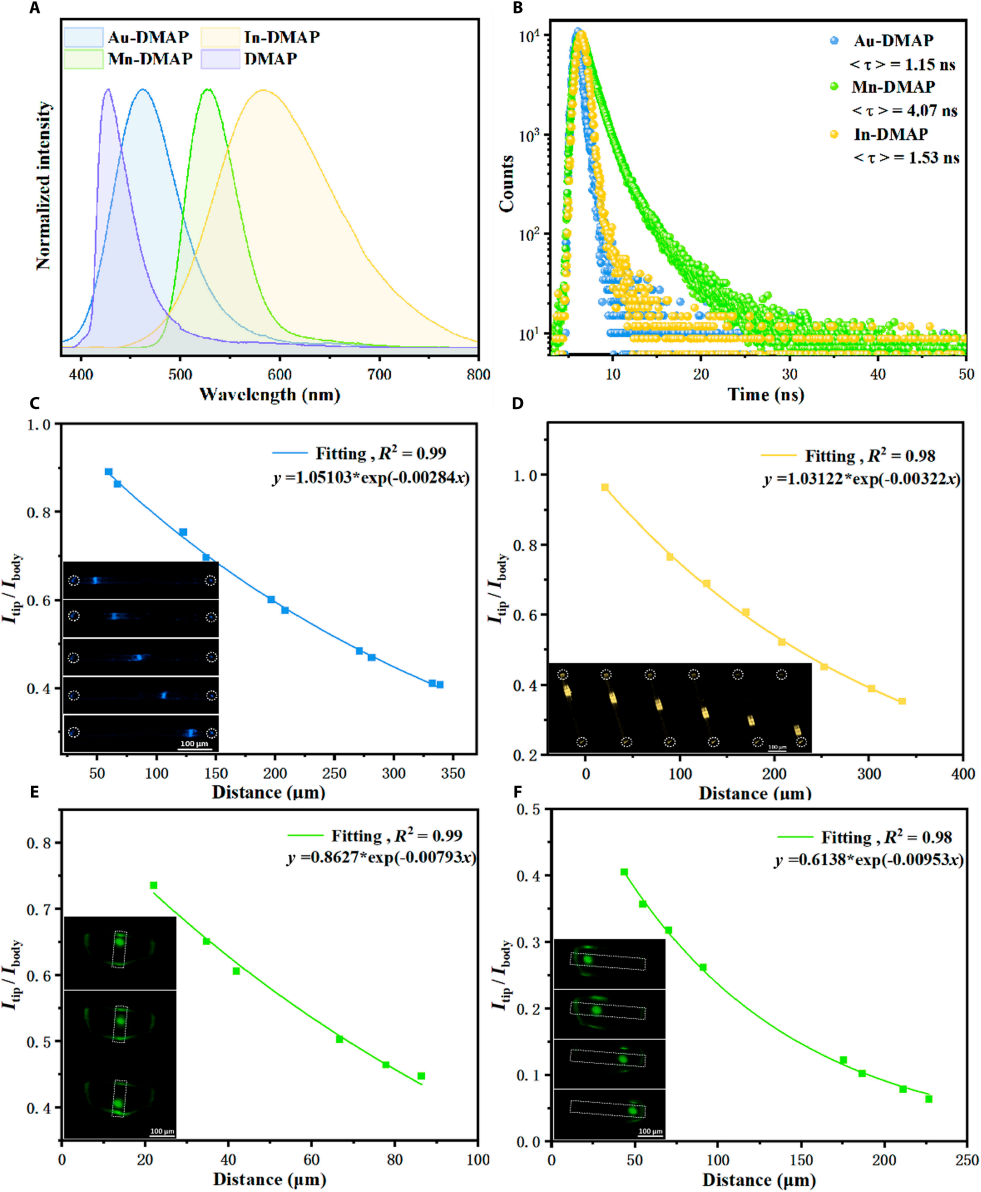
Luminescence and optical waveguide performance of Au-DMAP, Mn-DMAP, and In-DMAP. (A) PL spectra of Au-DMAP, Mn-DMAP, In-DMAP, and DMAP (λ_ex_ = 365 nm). (B) Time-resolved PL decay curves of Au-DMAP, Mn-DMAP, and In-DMAP. (C to F) The plots of the relative PL intensity *I*_WG_/*I*_EX_ against the waveguiding distance (C for Au-DMAP; D for In-DMAP; E and F for short and long directions of Mn-IMDC) (EX: excited spot; WG: waveguided spot). Inset: optical waveguide performances of Au-DMAP, Mn-DMAP, and In-DMAP obtained from the individual 1D microrod and individual 2D microplate by exciting with a laser beam.

The PL spectra of OMHs were measured under the excitation of 365 nm UV light (Fig. 5A). DMAP, Au-DMAP, Mn-DMAP, and In-DMAP have similar fluorescence shapes but different emission wavelengths with major peaks located at around 427, 462, 530, and 582 nm, respectively, suggesting that these 0D OMHs have similar luminescence mechanisms, but different energy levels involving CT from metal halides to organic ligands [60,61]. The progressive red shift of PL emission is attributed to the decreasing energy difference between the ground and the first excited states of the metal halide units with different metal-to-ligand CT (MLCT) and halogen-to-ligand CT (XLCT) processes [62–64]. The ultraviolet (UV)–vis absorption spectra (Fig. S6) reveal that the absorption intensity of Mn-DMAP around 350 nm is substantially higher than that of Au-DMAP and In-DMAP, which is consistent with the higher PLQY value of Mn-DMAP (Φ_f_ = 53%) than those of In-DMAP (Φ_f_ = 20%) and Au-DMAP (Φ_f_ = 4%). Correspondingly, the fluorescence lifetime of Mn-DMAP (4.07 ns, Table S5) is also longer than 2 other OMHs (1.15 ns for Au-DMAP and 1.53 ns for In-DMAP, Fig. 5B and Table S5).

To better understand the effect of electronic structures on the photoexcitation/emission properties, molecular orbitals and energy levels were obtained based on the density functional theory (DFT) and the time-dependent density functional theory (TDDFT) calculations. The graphs of molecular orbitals (Figs. S3 to S5) show that the highest occupied molecular orbitals of all 3 OMHs are located in the π orbitals of the metal halides, while the lowest unoccupied molecular orbitals are populated on the π* orbitals of the organic ligand DMAP, indicating that the excitations of all 3 0D OMHs are π–π* MLCT and XLCT singlet states. In addition, the gradual decrease of the calculated singlet state energy level difference (Table S3) is also close to those in the experimental PL spectra.

To explore the light propagation characteristics of the 1D microcrystals of Au-DMAP and In-DMAP OMHs, the optical waveguide performance was investigated. Spatially resolved PL imaging measurements were performed by locally exciting individual 1D microrods by using a focused laser beam (*λ*_ex_ = 375 nm). The OLC value was calculated based on the single-exponential formula *I*_tip_/*I*_body_ = *A*exp(−*αD*), where *I*_tip_ and *I*_body_ are the intensity of the waveguide emission and excitation points, D is the distance between these 2 points, and α is the OLC value [65,66]. Figure 5C and D demonstrate the micro-sized images, and the fitted OLC values of Au-DMAP and In-DMAP can be further obtained by shifting the laser point position on the microcrystals. The fitted OLC values of Au-DMAP and In-DMAP (*R*_α_ = 2.84 dB mm^−1^ and *R*_α_ = 3.22 dB mm^−1^) are lower than the majority of existing optical waveguide materials (including both inorganic and organic systems, Table S4). In particular, they are even lower than the recently reported inorganic–organic hybrid Au nanoclusters [25], indicating that these OMHs have a novel optical waveguide performance [67–71], which is beneficial for flexible photonic applications.

As for the Mn-DMAP microplate, it shows a typical 2D optical waveguide [72–74] (Fig. 5E and F). When the laser beam is focused on the center of Mn-DMAP, the generated photons are confined in the OMH microcrystal and all 4 edges of the 2D microplate exhibit photoemission, which decreases in intensity as the propagation distance increases. The OLC values (*R*_α_) of 7.93 and 9.53 dB mm^−1^ can be determined along the short-axis [100] and long-axis [001] directions of the crystal (Fig. 5E and F) respectively, indicating an obviously anisotropic 2D optical waveguide characteristic. Therefore, all 3 OMHs have the ability to transmit photons efficiently, which facilitates the design and synthesis of multi-port and color-tunable 1D and 2D optical waveguide materials.

### Synthesis and structural analysis of OMH heterojunctions

To solve the current problem of the single color and single port for conventional optical waveguides, we have further designed and fabricated 2 different heterojunctions based on the 3 OMHs. A multi-port optical waveguide output with heterostructure can be achieved by photoexcitation of trunk and branch microstructures (Fig. 1). Two crystalline heterojunctions consist of 1D Au-DMAP or In-DMAP and 2D Mn-DMAP microstructures, respectively. Both heterojunctions were prepared by a typical epitaxial growth method [75]. Firstly, the 1D microrods of Au-DMAP as trunks were grown by primary self-assembly. After that, the saturated solution of Mn-DMAP was dropped onto the Au-DMAP trunks, and the 2D Mn-DMAP microplates were epitaxially grown on the side surface of the microrods. The crystalline heterojunction 1 can be achieved after solution evaporation. Similarly, heterojunction 2 can be obtained by the epitaxial growth of Mn-DMAP 2D microplates on the side surface of In-DMAP trunk microrods.

**Fig. 6.**
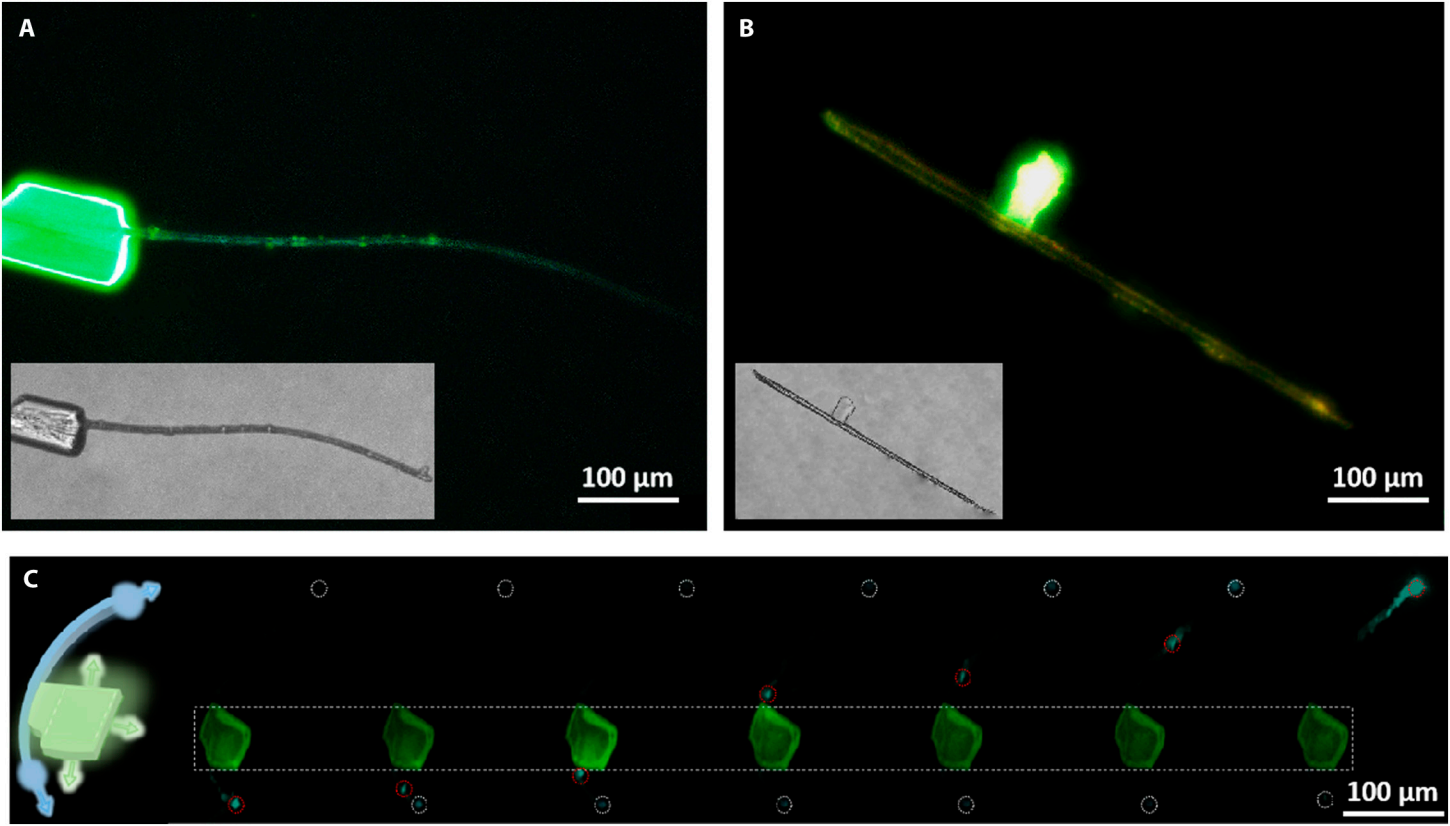
Optical characteristic of 2 heterojunctions. The fluorescence microscopy pictures of (A) heterojunction 1 and (B) heterojunction 2. Inset: microscopy pictures under bright field. (C) Optical waveguide performances of heterojunction 1 by exciting with a laser beam.

The fluorescence microscopy image of heterojunction 1 exhibits a bendable Au-DMAP microrod and the bright green edges of the Mn-DMAP microplate under unfocused UV excitation of a xenon lamp (Fig. 6A). It shows that the high flexibility of Au-DMAP and the high quantum yield of Mn-DMAP are simultaneously maintained in this heterostructure. Moreover, heterojunction 2 shows that In-DMAP and Mn-DMAP exhibit individual 1D yellow and 2D green optical waveguide, respectively, which confirms their high quantum yield for guaranteeing the color-tunable photonic output (Fig. 6B). Moreover, the local excitation of heterojunction 1 using a focused laser beam (*λ*_ex_ = 375 nm) further demonstrates that the photonic signal could transfer to 2 opposite directions around the 1D microrod and 2D microplate, respectively, confirming the feasibility of achieving multi-port and multi-color optical waveguide output by the formation of a heterostructure (Fig. 6C). The multiple components of the heterostructure ensure the ability to emit multi-color light simultaneously. In addition, by changing the excitation site, the luminescence color of each port will be changed due to the combined effect of optical waveguides and self-absorption. Therefore, the molecular self-assembly of the interconnection onto the highly flexible molecular crystals provides a new way for the development of heterostructure-based flexible optical waveguides with multi-site and space-resolved color-tunable photoemission at the micro/nanoscale.

**Fig. 7.**
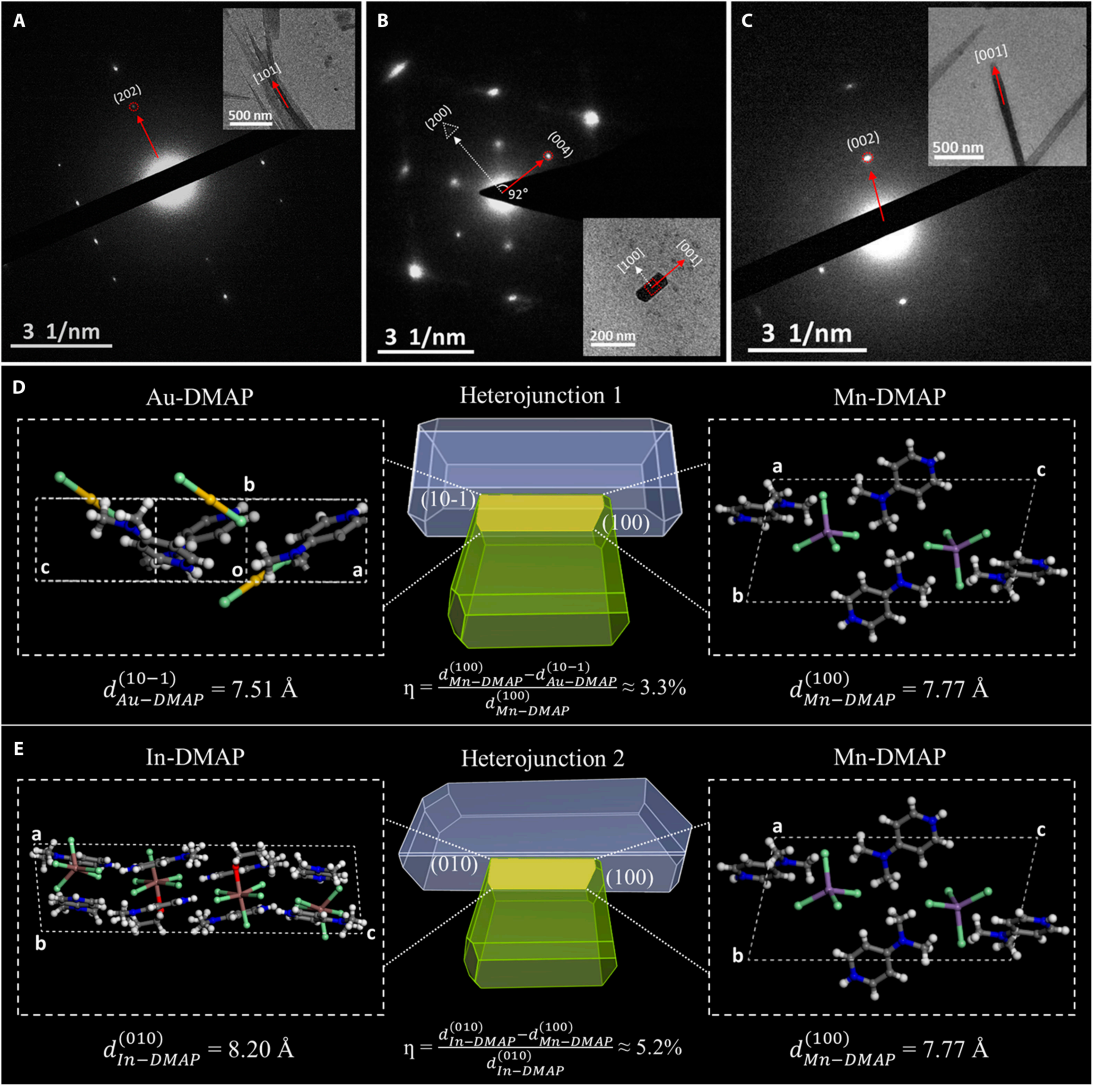
Selected-area electron diffraction patterns of the (A) Au-DMAP microrod, (B) Mn-DMAP microplate, and (C) In-DMAP microrod. Insets: the corresponding TEM images. (D and E) Morphology simulation calculation of 2 heterojunctions.

To better understand the assembly mechanism of 2 heterojunctions, a combination of selected-area electron diffraction and crystal morphology prediction has been performed. As shown in Fig. 7A to C, the Au-DMAP microrod grows along the [101] direction, which is consistent with the predominant growth orientation simulated by theoretical calculations. Similarly, the predominant growth orientation of the Mn-DMAP microplate is in the [001] direction with red arrows and the orthogonal [100] direction with white arrows; the predominant growth orientation of the In-DMAP microrod is along the [001] direction, which is consistent with the simulated results. The growth mechanism of crystalline heterostructures is generally considered as the surface selective growth on account of the low lattice mismatching. Lattice mismatch ratio *f* can be calculated by the equation *f* = (n_1_d_1_ − n_2_d_2_)/d_1_ (d_1_ and d_2_ are the intergranular spacing of the crystal planes of each heterojunction component in contact with each other, and n_1_ and n_2_ are the constants to make n_1_d_1_ numerically similar to n_2_d_2_) [76,77]. Generally, the criterion for the formation of heterojunction is tightly restricted such that *f* is less than 6% [78]. Considering the predominant growth orientations and crystal plane parameters of Au-DMAP, Mn-DMAP, and In-DMAP, we suggest that the crystal planes between the 1D microrod and 2D microplates in heterojunction 1 are the (10-1) crystal plane of Au-DMAP and the (100) crystal plane of Mn-DMAP (Fig. 7D), with an intergranular spacing of 7.51 Å and 7.77 Å, respectively. Therefore, the *f* value between Au-DMAP and Mn-DMAP at the interface can be calculated as low as 3.3%. The low lattice mismatch ratio is advantageous for the nucleation and epitaxial growth of Mn-DMAP on the side of Au-DMAP to form OMH heterojunctions. Correspondingly, the interface of heterojunction 2 is constructed by the (010) crystal plane of In-DMAP and the (100) crystal plane of Mn-DMAP (Fig. 7E), with an intergranular spacing of 8.20 Å and 7.77 Å, respectively, and an *f* value of 5.2%. This demonstrates that Mn-DMAP can self-assemble with In-DMAP to form the heterostructure. To verify the accuracy of the calculation, we have also performed powder x-ray diffraction (PXRD) simulations (Fig. S7), and the main characteristic peaks correspond well to the measured PXRD patterns in their crystal state. More crystal planes were exposed for the 3 crystals after grinding, which also agreed with the simulated XRD patterns and, thus, verified the accuracy of the simulated result.

## Discussion

In summary, beyond the traditional purely organic flexible crystals, new types of 0D OMHs have been facilely fabricated, in which Au-DMAP and In-DMAP microrods exhibit a highly elastic performance. The 3 OMHs present blue, green, and orange photoemission with the PLQY as high as 53%. DFT and TDDFT calculations indicate that the MLCT and XLCT processes in OMHs play a key role in the color-tunable luminescence. In particular, based on the theoretical analysis on intermolecular interactions, we demonstrate that the highly elastic properties of 2 flexible crystals can be achieved by 2 different mechanisms: herringbone structures and slip planes. In addition, both crystals exhibit excellent 1D waveguide performance with rather low OLCs, outperforming the state-of-the-art purely organic and inorganic systems. The Mn-DMAP microplate also exhibits excellent 2D optical waveguide, with obvious anisotropic optical loss in the growth direction [001] and epitaxial direction [100]. According to the low lattice mismatch ratio between the Mn-DMAP and Au-DMAP (3.3%), or between Mn-DMAP and In-DMAP (5.2%), 2 novel OMH crystalline heterojunctions with flexible optical waveguide and multiple photonic output ports have been further obtained. Extending the application prospects of optical waveguide materials in multi-channel and in complex environments, the integration of high elasticity and optical waveguide could also bring opportunities for complex smart photonic devices in the future.

## Materials and Methods

### Materials and reagents

All the reagents (DMAP, gold (I) chloride, manganese chloride, and indium chloride) were purchased from Sigma Chemistry Co. Ltd. and used without further purification.

### Synthesis of 0D OMHs

The crystalline Au-DMAP, Mn-DMAP, and In-DMAP samples were prepared via the hydrothermal method and solvent volatilization. Specifically, a mixture of AuCl or MnCl_2_ or InCl_3_ (1 mmol) with DMAP (1 mmol, 0.0611 g), 1 ml of HCl, and 7 ml of EtOH was ultrasonically mixed for 10 min. The mixture was then transferred to a Teflon reactor (25 ml) and heated at 100 °C for 48 h. Then, the mixture was cooled at a rate of 5 °C·h^−1^ to room temperature. Then, the solution was volatilized at room temperature until the colorless crystals were precipitated, filtered, and washed 3 times with water (3 × 10 ml).

### Instrumentation

SCXRD data of the samples were collected on an Oxford Diffraction SuperNova area-detector diffractometer using mirror optics monochromated Cu Kα radiation at room temperature. UV–vis absorption spectra were performed on a Shimadzu UV-3600 spectrophotometer at room temperature. PL microscope images of crystals were taken under an OLYMPUS IX71 fluorescence microscope. The optical waveguide tests were performed using a multidimensional confocal microfluorescence imaging system, which was imaged under the excitation of a laser beam at 375 nm and the OLCs were calculated. All the relevant PL tests including fluorescence and time-resolved PL lifetime were conducted on an Edinburgh FLS980 fluorescence spectrometer with the 365-nm excited wavelength, and the scanning speed was 1,200 nm/min. The time-resolved fluorescence decay spectroscopy was performed with a fixed-wavelength picosecond laser of 375 nm. The PLQY of the crystals were determined by using a Teflon-lined integrating sphere (F-M101, Edinburgh, diameter: 150 mm and weight: 2 kg) accessory in an FLS980 fluorescence spectrometer.

### Mechanical characterization

Mechanical tests of Au-DMAP and In-DMAP under external stress were collected on an OLYMPUS IXTI fluorescence microscope by pushing the crystal with a needle until it fractured out of the stress limit. The pictures of the fracture critical point were intercepted from the recorded video and modeled in a plane coordinate system. The crystals at the fracture moment were fitted to curves, the second-order derivatives and curvature were calculated by origin and searched for the maximum curvature, and the ultimate strain *ε_n_* was calculated for both crystals based on the calculated maximum curvature and the equation *ε_n_* = *d*/2*r* [79].

### Theoretical calculations

IGMH is a method of graphically presenting the interaction forces within a chemical system [80]. The geometries of ground state and excited states of Au-DMAP, Mn-DMAP, and In-DMAP were optimized by the DFT and the TDDFT, using the PBE0 functional with the 6-311G(d) basis set for C, H, and N, and the lanl2dz basis set for Au, Mn, and In. The D3 Grimme’s dispersion term with Becke–Johnson damping was added to the PBE0 functional to better describe the intermolecular non-covalent interactions. All works here were calculated by the D.01 revision of the Gaussian 09 program package [81] and analyzed by Multiwfn [82] and VMD [83].

### IGMH

A brief explanation of the IGMH diagram is given here: IGMH is the full name of the independent gradient model based on Hirshfeld division. As shown in Figs. 2C and 3C, the equivalent surface with a numerical ordinate within the inset corresponds to the strength of the intermolecular interaction at that site. The horizontal coordinates of the IGMH plot, sign(*λ*_2_)ρ, represent the type and strength of the interaction. The lower values of the horizontal coordinates represent stronger intermolecular gravitational forces, and the larger values of the horizontal coordinates represent stronger repulsive forces. The “g” seen in the vertical coordinate represents the gradient here, which is the sum of the electron density gradients of the 2 atoms or fragments of atoms. δ_g_ is defined as the sum of the absolute values of g minus g. Thus, the interaction between 2 atoms or fragments of 2 atoms can be expressed in a value of δ_g_, which is derived from the overlap of the densities of the 2 atoms or fragments and implies the existence of an interaction between the 2 atoms or fragments. The higher the density overlap between the 2 atoms, the higher the value corresponding to the interaction between the 2 atoms, which also means that the interaction between the atoms is stronger and thus brings the atoms closer together.

## Data Availability

All data in this study are available in this paper and there are no restrictions on the data availability.

## Supplementary Materials

Supplementary 1 Figs. S1 to S9 Tables S1 to S5 References [84–89]
