# Supplementary material for: Flexible Crystal Heterojunctions of Low-Dimensional Organic Metal Halides Enabling Color-Tunable Space-Resolved Optical Waveguides
Source: Research (Wash D C). 2023 Oct 30;6:0259. doi: 10.34133/research.0259 (PMC10616971; doi:10.34133/research.0259)

checkCIF/PLATON report

You have not supplied any structure factors. As a result the full set of tests cannot be run.

THIS REPORT IS FOR GUIDANCE ONLY. IF USED AS PART OF A REVIEW PROCEDURE FOR PUBLICATION, IT SHOULD NOT REPLACE THE EXPERTISE OF AN EXPERIENCED CRYSTALLOGRAPHIC REFEREE.

No syntax errors found. CIF dictionary Interpreting this report

Datablock: 7

Bond precision:	C-C = 0.0300 A		Wavelength=1.54178
Cell:	a=8.9119(2)	b=4.0797(1)	c=13.9529(2)
	alpha=90	beta=90	gamma=90
Temperature:	293 K		

	Calculated	Reported
Volume	507.298(18)	507.298(18)
Space group	P m n 21	P m n 21
Hall group	P 2ac -2	P 2ac -2
Moiety formula	C7 H11 N2, Au Cl2	?
Sum formula	C7 H11 Au Cl2 N2	C7 H11 Au Cl2 N2
Mr	391.05	391.04
Dx,g cm-3	2.560	2.560
Z	2	2
Mu (mm-1)	31.683	31.683
F000	360.0	360.0
F000'	353.32	
h,k,lmax	11,5,17	11,5,17
Nref	1126[587]	918
Tmin,Tmax	0.056,0.205	0.314,1.000
Tmin'	0.003	

Correction method= # Reported T Limits: Tmin=0.314 Tmax=1.000
AbsCorr = MULTI-SCAN

Data completeness= 1.56/0.82 Theta(max)= 75.939

R(reflections)= 0.0749(901) wR2(reflections)= 0.2062(918)

S = 1.055 Npar= 66

The following ALERTS were generated. Each ALERT has the format
test-name_ALERT_alert-type_alert-level.
Click on the hyperlinks for more details of the test.

Alert level B

```
PLAT342_ALERT_3_B Low Bond Precision on  C-C Bonds ...............       0.03 Ang.
```

Alert level C

```
RINTA01_ALERT_3_C  The value of Rint is greater than 0.12
            Rint given   0.129
STRVA01_ALERT_4_C           Flack test results are ambiguous.
           From the CIF: _refine_ls_abs_structure_Flack    0.460
           From the CIF: _refine_ls_abs_structure_Flack_su    0.100
PLAT020_ALERT_3_C The Value of Rint is Greater Than 0.12 .........      0.129 Report
PLAT220_ALERT_2_C NonSolvent   Resd 1  C   Ueq(max)/Ueq(min) Range        3.9 Ratio
PLAT242_ALERT_2_C Low    'MainMol' Ueq as Compared to Neighbors of         C3 Check
PLAT250_ALERT_2_C Large U3/U1 Ratio for Average U(i,j) Tensor ....        2.1 Note
```

Alert level G

```
PLAT003_ALERT_2_G Number of Uiso or Uij Restrained non-H Atoms ...          2 Report
PLAT007_ALERT_5_G Number of Unrefined Donor-H Atoms ..............          1 Report
PLAT186_ALERT_4_G The CIF-Embedded .res File Contains ISOR Records          1 Report
PLAT199_ALERT_1_G Reported _cell_measurement_temperature ..... (K)        293 Check
PLAT200_ALERT_1_G Reported   _diffrn_ambient_temperature ..... (K)        293 Check
PLAT304_ALERT_4_G Non-Integer Number of Atoms in ..... (Resd  2  )       1.50 Check
PLAT794_ALERT_5_G Tentative Bond Valency for Au2       (I)       .       0.95 Info
PLAT860_ALERT_3_G Number of Least-Squares Restraints .............         13 Note
PLAT883_ALERT_1_G No Info/Value for _atom_sites_solution_primary .     Please Do !
PLAT965_ALERT_2_G The SHELXL WEIGHT Optimisation has not Converged     Please Check
```

0 **ALERT level A** = Most likely a serious problem - resolve or explain
1 **ALERT level B** = A potentially serious problem, consider carefully
6 **ALERT level C** = Check. Ensure it is not caused by an omission or oversight
10 **ALERT level G** = General information/check it is not something unexpected

3 ALERT type 1 CIF construction/syntax error, inconsistent or missing data
5 ALERT type 2 Indicator that the structure model may be wrong or deficient
4 ALERT type 3 Indicator that the structure quality may be low
3 ALERT type 4 Improvement, methodology, query or suggestion
2 ALERT type 5 Informative message, check

It is advisable to attempt to resolve as many as possible of the alerts in all categories. Often the minor alerts point to easily fixed oversights, errors and omissions in your CIF or refinement strategy, so attention to these fine details can be worthwhile. In order to resolve some of the more serious problems it may be necessary to carry out additional measurements or structure refinements. However, the purpose of your study may justify the reported deviations and the more serious of these should normally be commented upon in the discussion or experimental section of a paper or in the "special_details" fields of the CIF. checkCIF was carefully designed to identify outliers and unusual parameters, but every test has its limitations and alerts that are not important in a particular case may appear. Conversely, the absence of alerts does not guarantee there are no aspects of the results needing attention. It is up to the individual to critically assess their own results and, if necessary, seek expert advice.

Publication of your CIF in IUCr journals

A basic structural check has been run on your CIF. These basic checks will be run on all CIFs submitted for publication in IUCr journals (*Acta Crystallographica*, *Journal of Applied Crystallography*, *Journal of Synchrotron Radiation*); however, if you intend to submit to *Acta Crystallographica Section C* or *E* or *IUCrData*, you should make sure that full publication checks are run on the final version of your CIF prior to submission.

Publication of your CIF in other journals

Please refer to the *Notes for Authors* of the relevant journal for any special instructions relating to CIF submission.

PLATON version of 19/02/2022; check.def file version of 19/02/2022

Datablock 7 - ellipsoid plot

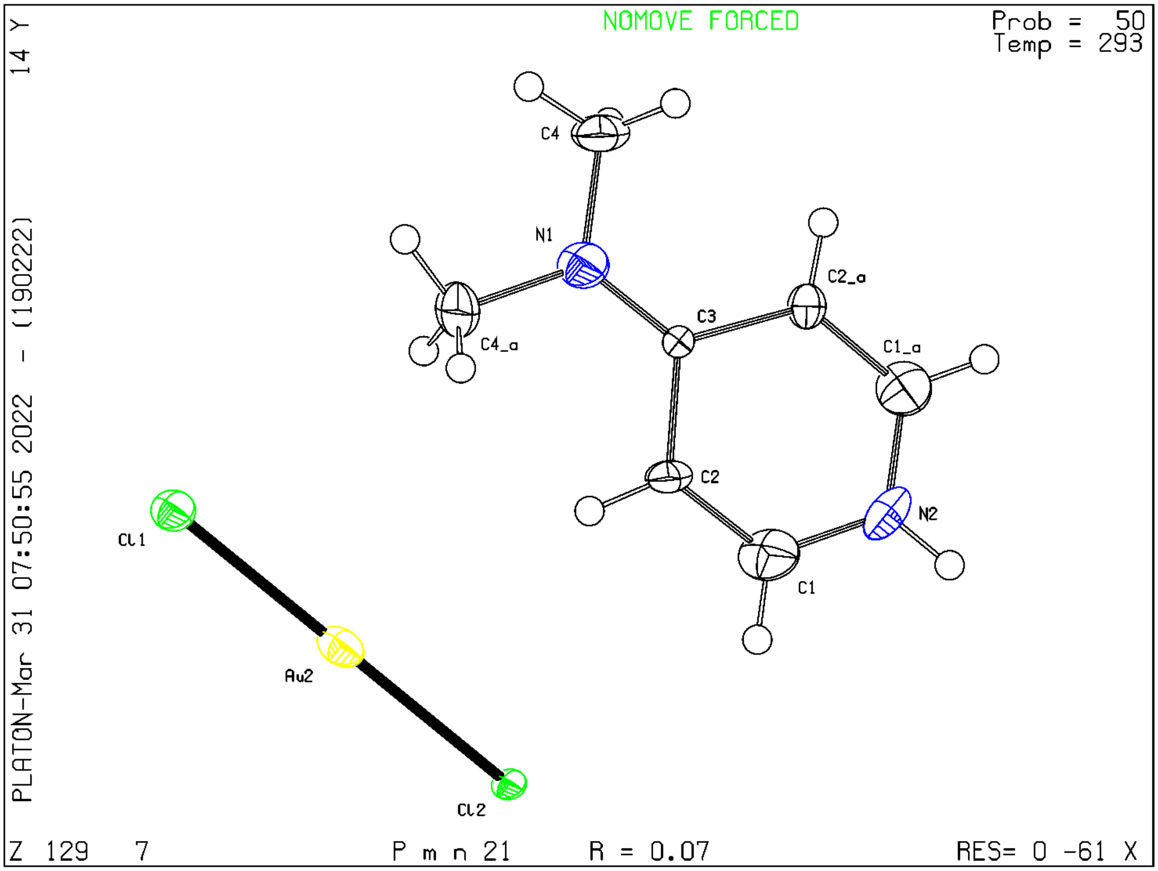

Supplement: Supplementary 1 — Figs. S1 to S9 Tables S1 to S5 References [84–89] [file research.0259.f1.zip › checkcif-Au-DMAP.pdf]
