# Supplementary material for: Flexible Crystal Heterojunctions of Low-Dimensional Organic Metal Halides Enabling Color-Tunable Space-Resolved Optical Waveguides
Source: Research (Wash D C). 2023 Oct 30;6:0259. doi: 10.34133/research.0259 (PMC10616971; doi:10.34133/research.0259)

checkCIF/PLATON report

Structure factors have been supplied for datablock(s) 20221004a_auto

THIS REPORT IS FOR GUIDANCE ONLY. IF USED AS PART OF A REVIEW PROCEDURE FOR PUBLICATION, IT SHOULD NOT REPLACE THE EXPERTISE OF AN EXPERIENCED CRYSTALLOGRAPHIC REFEREE.

No syntax errors found. CIF dictionary Interpreting this report

Datablock: 20221004a_auto

Bond precision: C-C = 0.0125 A Wavelength=1.54184

Cell:	a=7.1204(3)	b=12.2184(5)	c=25.4421(7)
	alpha=90.381(3)	beta=95.114(3)	gamma=99.500(3)
Temperature:	100 K		

	Calculated	Reported
Volume	2173.90(14)	2173.90(14)
Space group	P -1	P -1
Hall group	-P 1	-P 1
Moiety formula	C2 H6 Cl5 In O, 4(C7 H11 N2), Cl5 In	C2 H6 Cl5 In O, Cl5 In, 4(C7 H11 N2)
Sum formula	C30 H50 Cl10 In2 N8 O	C30 H50 Cl10 In2 N8 O
Mr	1122.92	1122.92
Dx,g cm-3	1.715	1.715
Z	2	2
Mu (mm-1)	14.427	14.427
F000	1124.0	1124.0
F000'	1133.68	
h,k,lmax	8,15,32	8,15,31
Nref	9150	8603
Tmin,Tmax	0.221,0.486	0.317,1.000
Tmin'	0.092	

Correction method= # Reported T Limits: Tmin=0.317 Tmax=1.000
AbsCorr = MULTI-SCAN

Data completeness= 0.940 Theta(max)= 76.771

R(reflections)= 0.0692(7275) wR2(reflections)= 0.1986(8603)

S = 1.100 Npar= 473

The following ALERTS were generated. Each ALERT has the format
test-name_ALERT_alert-type_alert-level.
Click on the hyperlinks for more details of the test.

Alert level C

```
PLAT042_ALERT_1_C Calc. and Reported MoietyFormula Strings  Differ     Please Check
PLAT342_ALERT_3_C Low Bond Precision on  C-C Bonds ..............    0.01247 Ang.
PLAT906_ALERT_3_C Large K Value in the Analysis of Variance ......     14.080 Check
PLAT906_ALERT_3_C Large K Value in the Analysis of Variance ......      2.738 Check
PLAT911_ALERT_3_C Missing FCF Refl Between Thmin & STh/L=    0.600         76 Report
PLAT934_ALERT_3_C Number of (Iobs-Icalc)/Sigma(W) > 10 Outliers ..          1 Check
PLAT971_ALERT_2_C Check Calcd Resid. Dens.  1.01Ang From In2             2.09 eA-3
PLAT971_ALERT_2_C Check Calcd Resid. Dens.  0.43Ang From Cl8             2.04 eA-3
PLAT971_ALERT_2_C Check Calcd Resid. Dens.  1.39Ang From In2             2.01 eA-3
PLAT971_ALERT_2_C Check Calcd Resid. Dens.  0.28Ang From Cl8             2.01 eA-3
PLAT971_ALERT_2_C Check Calcd Resid. Dens.  1.03Ang From In2             2.00 eA-3
PLAT971_ALERT_2_C Check Calcd Resid. Dens.  1.35Ang From In2             1.81 eA-3
PLAT971_ALERT_2_C Check Calcd Resid. Dens.  0.60Ang From Cl8             1.80 eA-3
PLAT971_ALERT_2_C Check Calcd Resid. Dens.  1.35Ang From In1             1.80 eA-3
PLAT971_ALERT_2_C Check Calcd Resid. Dens.  1.09Ang From In1             1.65 eA-3
PLAT971_ALERT_2_C Check Calcd Resid. Dens.  1.29Ang From O13             1.62 eA-3
PLAT972_ALERT_2_C Check Calcd Resid. Dens.  0.65Ang From In2            -2.31 eA-3
PLAT972_ALERT_2_C Check Calcd Resid. Dens.  0.79Ang From In2            -1.80 eA-3
PLAT972_ALERT_2_C Check Calcd Resid. Dens.  0.63Ang From In1            -1.64 eA-3
PLAT972_ALERT_2_C Check Calcd Resid. Dens.  0.70Ang From In1            -1.55 eA-3
PLAT977_ALERT_2_C Check Negative Difference Density on H9C       .      -0.42 eA-3
PLAT977_ALERT_2_C Check Negative Difference Density on H15A      .      -0.37 eA-3
PLAT977_ALERT_2_C Check Negative Difference Density on H25       .      -0.38 eA-3
```

Alert level G

```
PLAT002_ALERT_2_G Number of Distance or Angle Restraints on AtSite          4 Note
PLAT007_ALERT_5_G Number of Unrefined Donor-H Atoms .............          4 Report
PLAT083_ALERT_2_G SHELXL Second Parameter in WGHT  Unusually Large      27.48 Why ?
PLAT154_ALERT_1_G The s.u.'s on the Cell Angles are Equal ..(Note)      0.003 Degree
PLAT172_ALERT_4_G The CIF-Embedded .res File Contains DFIX Records          1 Report
PLAT173_ALERT_4_G The CIF-Embedded .res File Contains DANG Records          2 Report
PLAT232_ALERT_2_G Hirshfeld Test Diff (M-X)  In1      --Cl3      .        8.9 s.u.
PLAT794_ALERT_5_G Tentative Bond Valency for In1       (III)     .       3.04 Info
PLAT794_ALERT_5_G Tentative Bond Valency for In2       (III)     .       3.04 Info
PLAT860_ALERT_3_G Number of Least-Squares Restraints ............          3 Note
PLAT883_ALERT_1_G No Info/Value for _atom_sites_solution_primary .     Please Do !
PLAT910_ALERT_3_G Missing # of FCF Reflection(s) Below Theta(Min).          1 Note
PLAT912_ALERT_4_G Missing # of FCF Reflections Above STh/L=  0.600        467 Note
PLAT933_ALERT_2_G Number of HKL-OMIT Records in Embedded .res File         50 Note
PLAT941_ALERT_3_G Average HKL Measurement Multiplicity ..........         2.8 Low
PLAT978_ALERT_2_G Number C-C Bonds with Positive Residual Density.          0 Info
```

0 **ALERT level A** = Most likely a serious problem - resolve or explain
0 **ALERT level B** = A potentially serious problem, consider carefully
23 **ALERT level C** = Check. Ensure it is not caused by an omission or oversight
16 **ALERT level G** = General information/check it is not something unexpected

3 ALERT type 1 CIF construction/syntax error, inconsistent or missing data

```
 22 ALERT type 2 Indicator that the structure model may be wrong or deficient
  8 ALERT type 3 Indicator that the structure quality may be low
  3 ALERT type 4 Improvement, methodology, query or suggestion
  3 ALERT type 5 Informative message, check
```

It is advisable to attempt to resolve as many as possible of the alerts in all categories. Often the minor alerts point to easily fixed oversights, errors and omissions in your CIF or refinement strategy, so attention to these fine details can be worthwhile. In order to resolve some of the more serious problems it may be necessary to carry out additional measurements or structure refinements. However, the purpose of your study may justify the reported deviations and the more serious of these should normally be commented upon in the discussion or experimental section of a paper or in the "special_details" fields of the CIF. checkCIF was carefully designed to identify outliers and unusual parameters, but every test has its limitations and alerts that are not important in a particular case may appear. Conversely, the absence of alerts does not guarantee there are no aspects of the results needing attention. It is up to the individual to critically assess their own results and, if necessary, seek expert advice.

Publication of your CIF in IUCr journals

A basic structural check has been run on your CIF. These basic checks will be run on all CIFs submitted for publication in IUCr journals (*Acta Crystallographica*, *Journal of Applied Crystallography*, *Journal of Synchrotron Radiation*); however, if you intend to submit to *Acta Crystallographica Section C* or *E* or *IUCrData*, you should make sure that full publication checks are run on the final version of your CIF prior to submission.

Publication of your CIF in other journals

Please refer to the *Notes for Authors* of the relevant journal for any special instructions relating to CIF submission.

PLATON version of 12/09/2022; check.def file version of 09/08/2022

Datablock 20221004a_auto - ellipsoid plot

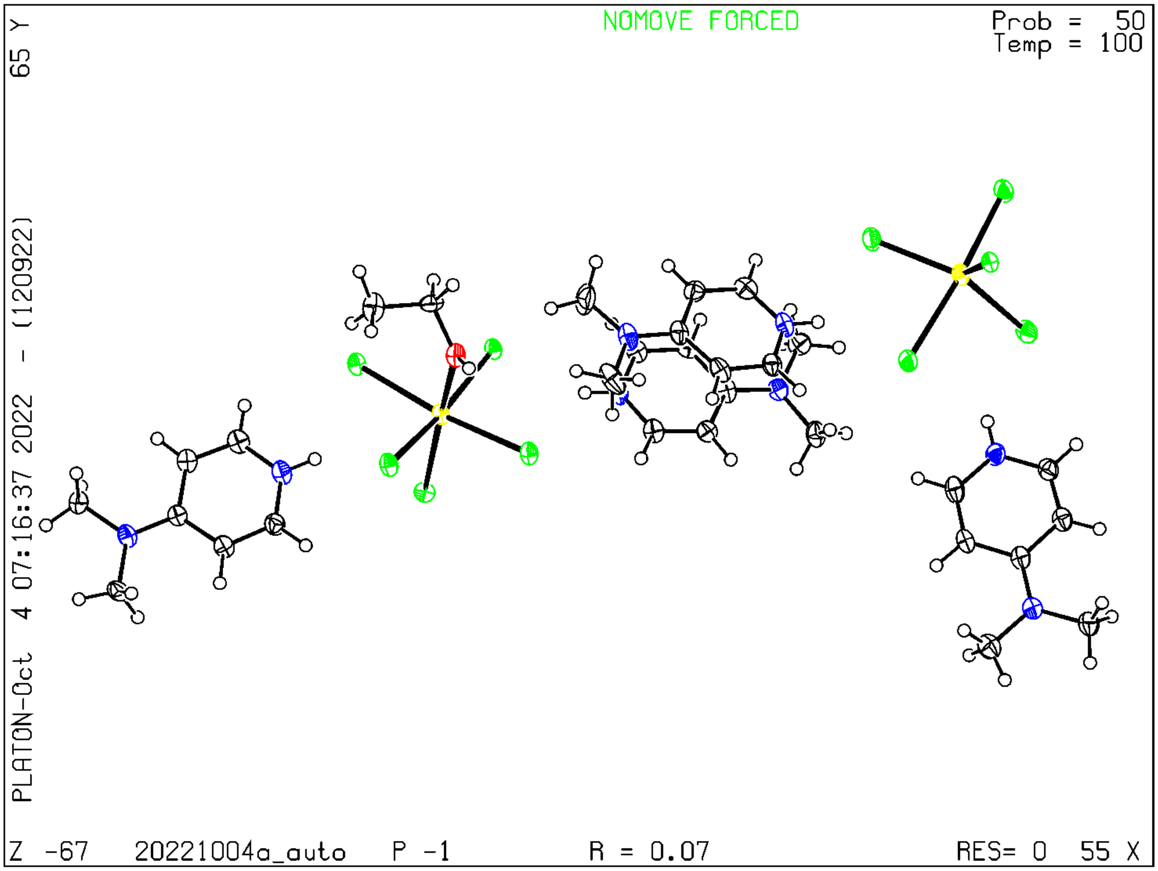

Supplement: Supplementary 1 — Figs. S1 to S9 Tables S1 to S5 References [84–89] [file research.0259.f1.zip › checkcif-In-DMAP.pdf]
