# Supplementary material for: Flexible Crystal Heterojunctions of Low-Dimensional Organic Metal Halides Enabling Color-Tunable Space-Resolved Optical Waveguides
Source: Research (Wash D C). 2023 Oct 30;6:0259. doi: 10.34133/research.0259 (PMC10616971; doi:10.34133/research.0259)

checkCIF/PLATON report

Structure factors have been supplied for datablock(s) 20220409a_auto

THIS REPORT IS FOR GUIDANCE ONLY. IF USED AS PART OF A REVIEW PROCEDURE FOR PUBLICATION, IT SHOULD NOT REPLACE THE EXPERTISE OF AN EXPERIENCED CRYSTALLOGRAPHIC REFEREE.

No syntax errors found. CIF dictionary Interpreting this report

Datablock: 20220409a_auto

Bond precision: C-C = 0.0085 A Wavelength=1.54184

Cell:	a=7.9851(3)	b=8.0431(4)	c=16.8411(6)
	alpha=101.752(4)	beta=95.394(3)	gamma=94.666(4)
Temperature:	301 K		

	Calculated	Reported
Volume	1048.64(8)	1048.63(8)
Space group	P -1	P -1
Hall group	-P 1	-P 1
Moiety formula	2(C7 H11 N2), Cl4 Mn	Cl4 Mn, 2(C7 H11 N2)
Sum formula	C14 H22 Cl4 Mn N4	C14 H20 Cl4 Mn N4
Mr	443.10	441.08
Dx,g cm-3	1.403	1.397
Z	2	2
Mu (mm-1)	9.827	9.827
F000	454.0	450.0
F000'	456.68	
h,k,lmax	10,10,21	10,10,21
Nref	4460	4160
Tmin,Tmax	0.213,0.374	0.379,1.000
Tmin'	0.112	

Correction method= # Reported T Limits: Tmin=0.379 Tmax=1.000
AbsCorr = MULTI-SCAN

Data completeness= 0.933 Theta(max)= 77.401

R(reflections)= 0.0714(3300) wR2(reflections)= 0.2236(4160)

S = 1.095 Npar= 212

The following ALERTS were generated. Each ALERT has the format

test-name_ALERT_alert-type_alert-level.

Click on the hyperlinks for more details of the test.

Alert level C

```
PLAT041_ALERT_1_C Calc. and Reported SumFormula    Strings  Differ     Please Check
PLAT042_ALERT_1_C Calc. and Reported MoietyFormula Strings  Differ     Please Check
PLAT043_ALERT_1_C Calculated and Reported Mol. Weight Differ by ..       2.02 Check
PLAT068_ALERT_1_C Reported F000 Differs from Calcd (or Missing)...     Please Check
PLAT094_ALERT_2_C Ratio of Maximum / Minimum Residual Density ....       2.71 Report
PLAT242_ALERT_2_C Low    'MainMol' Ueq as Compared to Neighbors of        C18 Check
PLAT242_ALERT_2_C Low    'MainMol' Ueq as Compared to Neighbors of        Mn1 Check
PLAT341_ALERT_3_C Low Bond Precision on  C-C Bonds ..............     0.0085 Ang.
PLAT767_ALERT_4_C INS Embedded LIST 6 Instruction Should be LIST 4     Please Check
PLAT906_ALERT_3_C Large K Value in the Analysis of Variance ......      3.912 Check
PLAT911_ALERT_3_C Missing FCF Refl Between Thmin & STh/L=    0.600         25 Report
PLAT934_ALERT_3_C Number of (Iobs-Icalc)/Sigma(W) > 10 Outliers ..          1 Check
```

Alert level G

```
FORMU01_ALERT_1_G  There is a discrepancy between the atom counts in the
            _chemical_formula_sum and _chemical_formula_moiety. This is
            usually due to the moiety formula being in the wrong format.
            Atom count from _chemical_formula_sum:   C14 H20 Cl4 Mn1 N4
            Atom count from _chemical_formula_moiety:C14 H22 Cl4 Mn1 N4
FORMU01_ALERT_2_G  There is a discrepancy between the atom counts in the
            _chemical_formula_sum and the formula from the _atom_site* data.
            Atom count from _chemical_formula_sum:C14 H20 Cl4 Mn1 N4
            Atom count from the _atom_site data:  C14 H22 Cl4 Mn1 N4
CELLZ01_ALERT_1_G Difference between formula and atom_site contents detected.
CELLZ01_ALERT_1_G ALERT: Large difference may be due to a
            symmetry error - see SYMMG tests
           From the CIF: _cell_formula_units_Z    2
           From the CIF: _chemical_formula_sum  C14 H20 Cl4 Mn N4
           TEST: Compare cell contents of formula and atom_site data

           atom    Z*formula  cif sites diff
           C         28.00     28.00    0.00
           H         40.00     44.00   -4.00
           Cl         8.00      8.00    0.00
           Mn         2.00      2.00    0.00
           N          8.00      8.00    0.00
PLAT007_ALERT_5_G Number of Unrefined Donor-H Atoms .............          2 Report
PLAT072_ALERT_2_G SHELXL First  Parameter in WGHT  Unusually Large       0.13 Report
PLAT794_ALERT_5_G Tentative Bond Valency for Mn1       (II)      .       2.11 Info
PLAT910_ALERT_3_G Missing # of FCF Reflection(s) Below Theta(Min).          1 Note
PLAT912_ALERT_4_G Missing # of FCF Reflections Above STh/L=  0.600        274 Note
PLAT933_ALERT_2_G Number of HKL-OMIT Records in Embedded .res File          3 Note
PLAT941_ALERT_3_G Average HKL Measurement Multiplicity ..........         2.7 Low
PLAT978_ALERT_2_G Number C-C Bonds with Positive Residual Density.          0 Info
```

0 **ALERT level A** = Most likely a serious problem - resolve or explain

0 **ALERT level B** = A potentially serious problem, consider carefully

12 **ALERT level C** = Check. Ensure it is not caused by an omission or oversight

```
12 ALERT level G = General information/check it is not something unexpected

 7 ALERT type 1 CIF construction/syntax error, inconsistent or missing data
 7 ALERT type 2 Indicator that the structure model may be wrong or deficient
 6 ALERT type 3 Indicator that the structure quality may be low
 2 ALERT type 4 Improvement, methodology, query or suggestion
 2 ALERT type 5 Informative message, check
```

It is advisable to attempt to resolve as many as possible of the alerts in all categories. Often the minor alerts point to easily fixed oversights, errors and omissions in your CIF or refinement strategy, so attention to these fine details can be worthwhile. In order to resolve some of the more serious problems it may be necessary to carry out additional measurements or structure refinements. However, the purpose of your study may justify the reported deviations and the more serious of these should normally be commented upon in the discussion or experimental section of a paper or in the "special_details" fields of the CIF. checkCIF was carefully designed to identify outliers and unusual parameters, but every test has its limitations and alerts that are not important in a particular case may appear. Conversely, the absence of alerts does not guarantee there are no aspects of the results needing attention. It is up to the individual to critically assess their own results and, if necessary, seek expert advice.

Publication of your CIF in IUCr journals

A basic structural check has been run on your CIF. These basic checks will be run on all CIFs submitted for publication in IUCr journals (*Acta Crystallographica*, *Journal of Applied Crystallography*, *Journal of Synchrotron Radiation*); however, if you intend to submit to *Acta Crystallographica Section C* or *E* or *IUCrData*, you should make sure that full publication checks are run on the final version of your CIF prior to submission.

Publication of your CIF in other journals

Please refer to the *Notes for Authors* of the relevant journal for any special instructions relating to CIF submission.

PLATON version of 10/05/2023; check.def file version of 10/05/2023

Datablock 20220409a_auto - ellipsoid plot

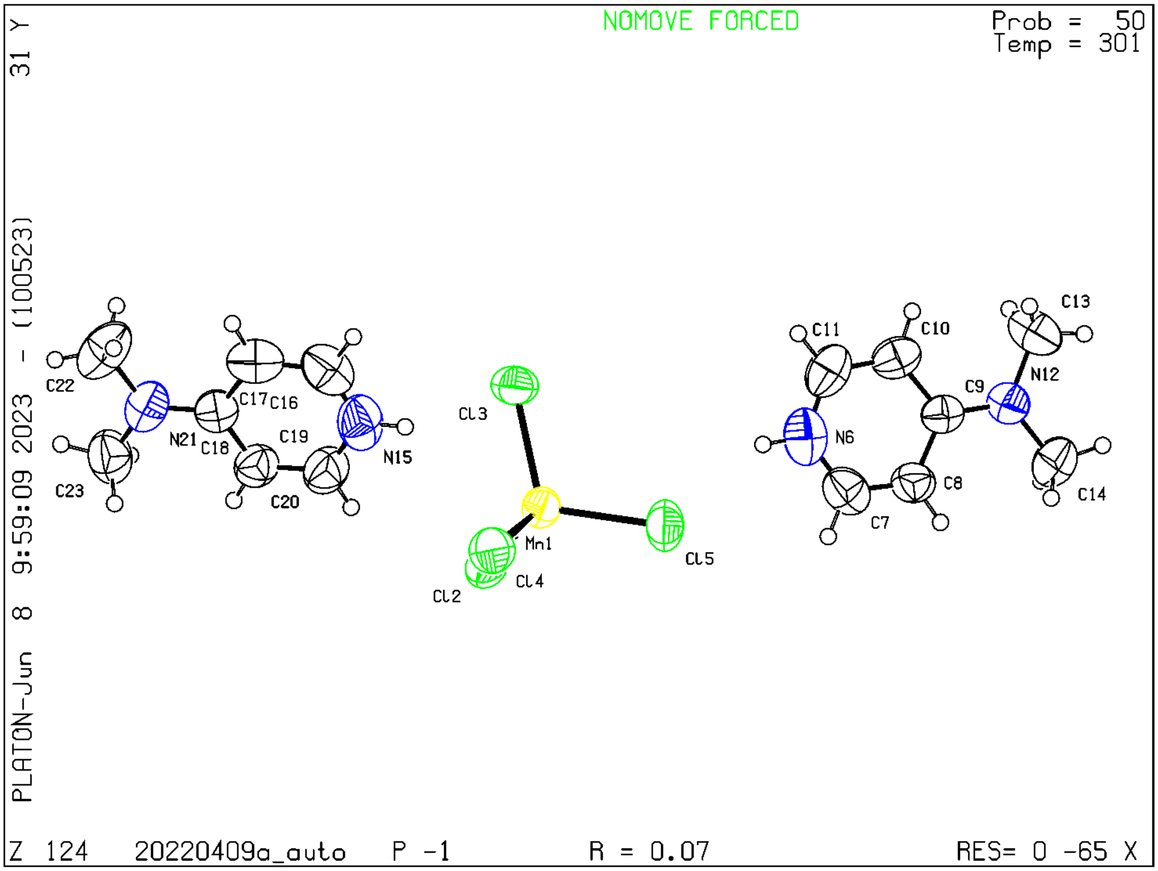

Supplement: Supplementary 1 — Figs. S1 to S9 Tables S1 to S5 References [84–89] [file research.0259.f1.zip › checkcif-Mn-DMAP.pdf]
