# Supplementary material for: Flexible Crystal Heterojunctions of Low-Dimensional Organic Metal Halides Enabling Color-Tunable Space-Resolved Optical Waveguides
Source: Research (Wash D C). 2023 Oct 30;6:0259. doi: 10.34133/research.0259 (PMC10616971; doi:10.34133/research.0259)
Supplement: Supplementary 1 — Figs. S1 to S9 Tables S1 to S5 References [84–89] [file research.0259.f1.zip › Supporting Materials.docx]

Supplementary Materials

*
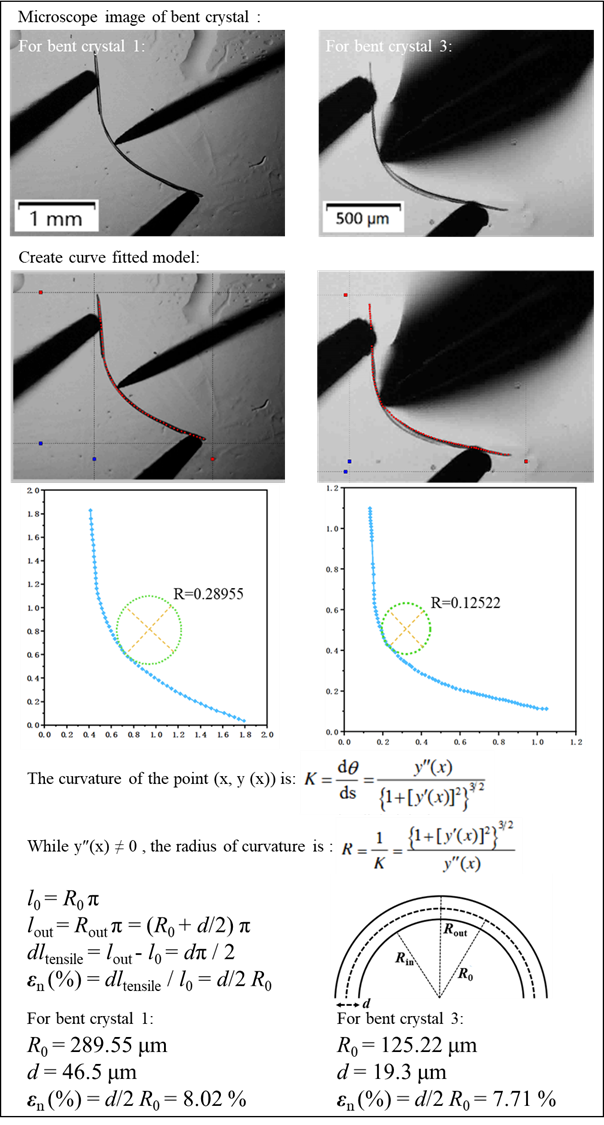
*

Fig. S1. Bending test and calculation of strain ability of two flexible materials Au-DMAP and In-DMAP.


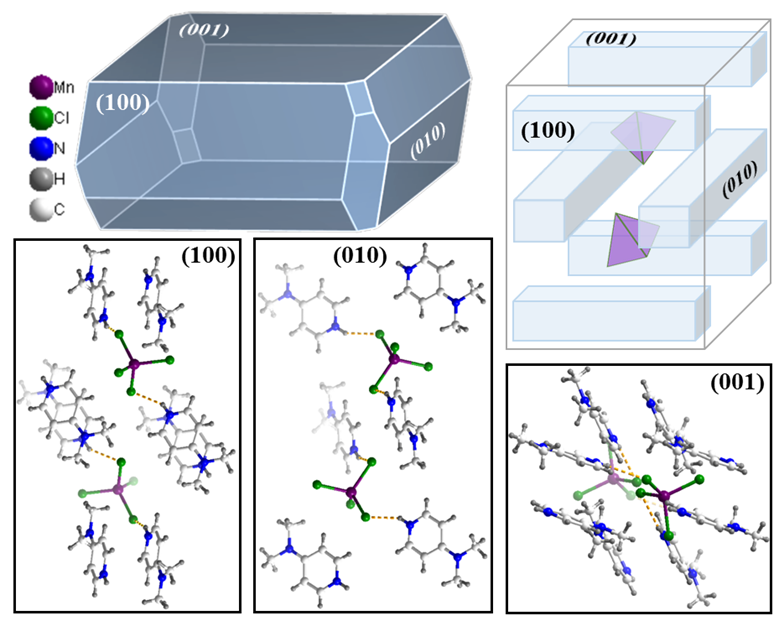


Fig. S2. Crystal structure of Mn-DMAP and illustration of crystal morphology based on calculation.


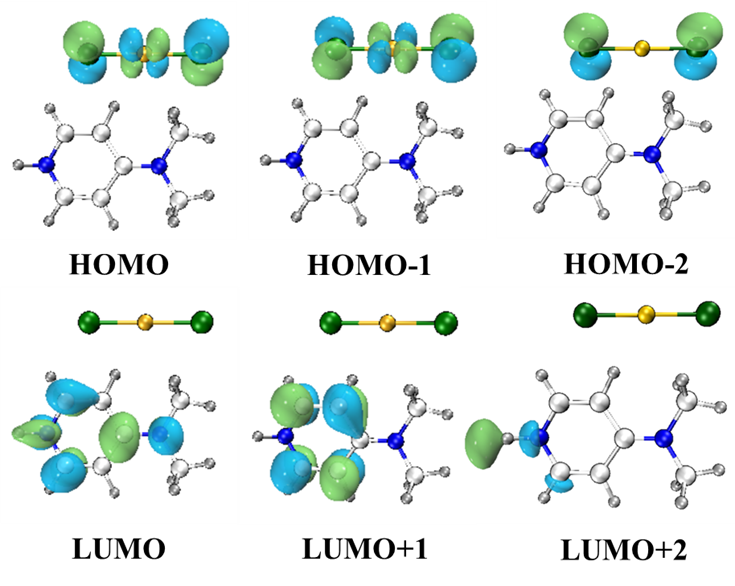


Fig. S3. The calculated molecular orbitals of Au-DMAP.


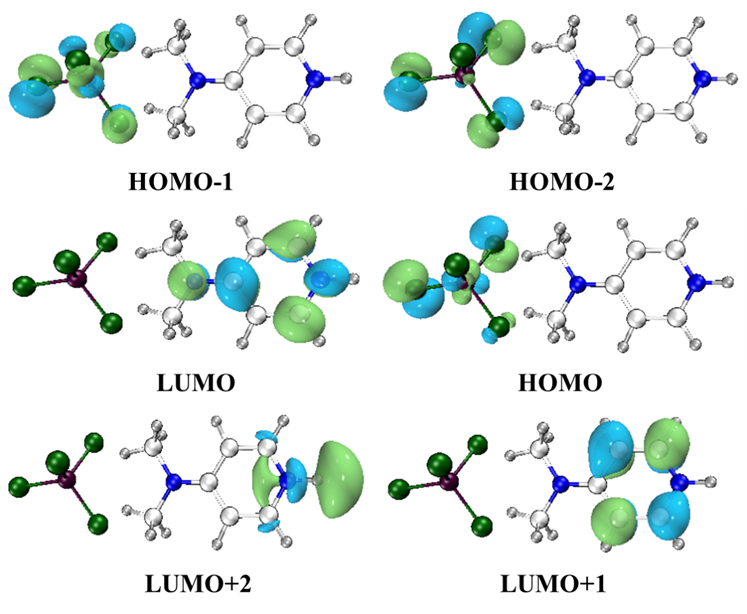


Fig. S4. The calculated molecular orbitals of Mn-DMAP.


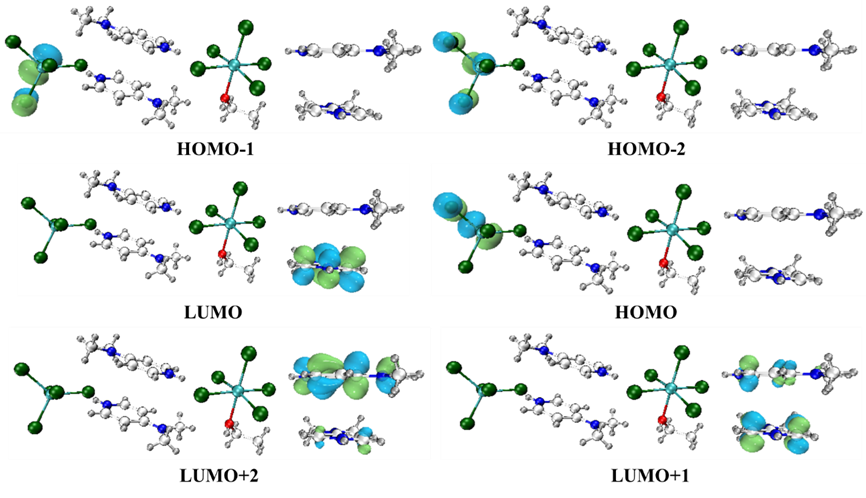


Fig. S5. The calculated molecular orbitals of In-DMAP.


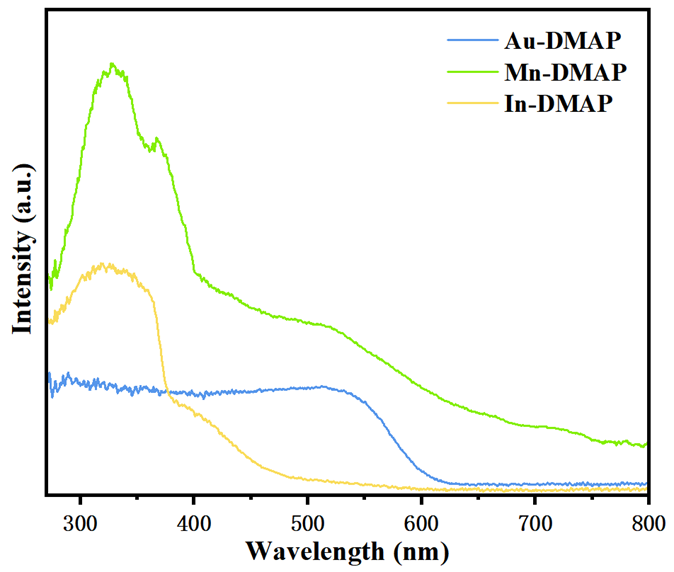


Fig. S6. UV-vis absorption spectra of Au-DMAP, In-DMAP and Mn-DMAP.


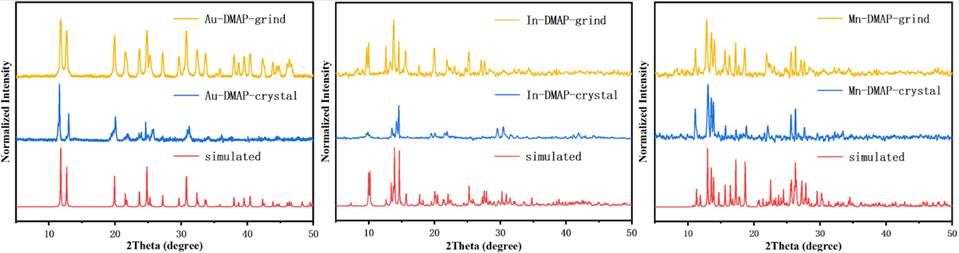


Fig. S7. PXRD patterns of Au-DMAP, In-DMAP and Mn-DMAP (crystalline, after grind and simulated).


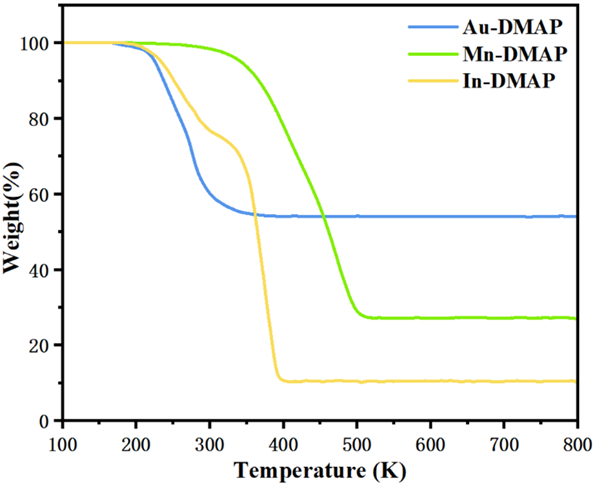


Fig. S8. TGA curves of Au-DMAP, In-DMAP and Mn-DMAP (crystalline, after grind and simulated).


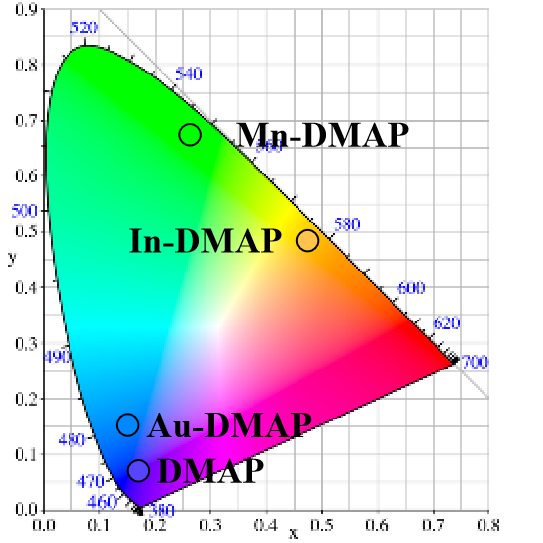


Fig. S9. CIE chromaticity diagram of Au-DMAP, In-DMAP and Mn-DMAP.

Table S1. Crystal data and structure refinement for Au-DMAP, Mn-DMAP and In-DMAP.

| **Samples** | **Au-DMAP**  **(CCDC: 2233211)** | **Mn-DMAP**  **(CCDC: 2233215)** | **In-DMAP**  **(CCDC: 2233217)** |
| --- | --- | --- | --- |
| **Formula** | C_7_H_11_AuCl_2_N_2_ | C_14_H_20_Cl_4_MnN_4_ | C_30_H_50_Cl_10_In_2_N_8_O |
| ***Mr*** | 391.05 | 441.08 | 1122.92 |
| **Temperature (K)** | 100.00(13) | 100.00(10) | 100.00(10) |
| **Crystal system** | Orthorhombic | Triclinic | Triclinic |
| **Space group** | Pmn2_1_ | P 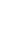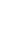 | P 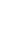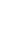 |
| **Crystal size (mm)** | 0.20 × 0.05 × 0.03 | 0.15 × 0.10 × 0.10 | 0.10 × 0.03 × 0.02 |
| ***a* (Å)** | 8.9119(2) | 7.9851(3) | 7.1204(3) |
| ***b* (Å)** | 4.0797(1) | 8.0431(4) | 12.2184(5) |
| ***c* (Å)** | 13.9529(2) | 16.8411(6) | 25.4421(7) |
| ***α* (°)** | 90 | 101.752(4) | 90.381(3) |
| ***β* (°)** | 90 | 95.394(3) | 95.114(3) |
| ***γ* (°)** | 90 | 94.666(4) | 99.500(3) |
| ***V*(Å^3^)** | 507.298(18) | 1048.63(8) | 2173.90(14) |
| ***Z*** | 2 | 2 | 2 |
| ***D*_calc_ (mg/m^3^)** | 2.560 | 1.397 | 1.715 |
| **θ Range (°)** | 5.891-75.939 | 5.401-77.401 | 3.489-76.771 |
| **F (000)** | 360.0 | 450.0 | 1124.0 |
| **Data/restraint/**  **parameters** | 918 / 13 / 66 | 4160 / 0 / 212 | 8603 / 3 / 473 |
| **Reflections collected** | 4416 | 11294 | 24253 |
| **Independent reflections** | 918 | 4160 | 8603 |
| **Goodness-of-fit on F^2^** | 1.055 | 1.096 | 1.100 |
| ***R*_int_** | 0.1290 | 0.0433 | 0.0786 |
| ***R*_1_[*I*>2σ(*І*)]** | 0.0749 | 0.0714 | 0.0692 |
| ***wR*_2_[*I*>2σ(*І*)]** | 0.2050 | 0.2123 | 0.1818 |
| ***R*_1_(all data)** | 0.0753 | 0.0838 | 0.0877 |
| ***wR*_2_(all data)** | 0.2062 | 0.2237 | 0.1986 |
| **Residuals(e Å^-3^)** | 4.453, -3.502 | 1.395, -0.514 | 2.006, -2.102 |

***R*_1_ = Σ||F_o_| – |F_c_||/Σ|F_o_|, *wR*_2_ = [Σ_w_(F_o_^2^ – F_c_^2^)^2^/Σ*w*(F_o_^2^)^2^]^1/2^**

Table S2. Lengths (Å) and angles (o) of N-H…Cl interactions for In-DMAP (100 K).

| **N-H…Cl interactions** | **lengths (Å)** | **angle** | **angle (o)** |
| --- | --- | --- | --- |
| **Cl(5)-H(5)** | 2.6488(16) | **Cl(5)-H(5)-N(5)** | 136.383(491) |
|  |  | **In(1)-Cl(5)-H5)** | 90.272(49) |
| **Cl(6)-H(3)** | 2.5105(20) | **Cl(6)-H(3)-N(3)** | 143.790(546) |
|  |  | **In(2)-Cl(6)-H(3)** | 97.484(73) |
| **Cl(10)-H(7)** | 2.4255(21) | **Cl(10)-H(7)-N(7)** | 145.876(505) |
|  |  | **In(2)-Cl(10)-H(7)** | 98.040(72) |
| **Cl(12)-H(5)** | 2.5410(21) | **Cl(12)-H(5)-N(5)** | 136.634(496) |
|  |  | **In(1)-Cl(12)-H(5)** | 93.997(69) |
| **Cl(13)-H(1)** | 2.5766(20) | **Cl(13)-H(1)-N(1)** | 134.883(521) |
|  |  | **In(1)-Cl(13)-H(1)** | 82.856(63) |

Table S3. Part of the singlet excited state transition configurations obtained by TD-DFT calculations and experimental emission wavelength of Au-DMAP, Mn-DMAP and In-DMAP.

| **organic metal halide hybrids** | **S_n_** | **Excitation (Coefficient>0.3)** | **Vertical excitation energy / eV** | **Vertical excitation wavelength / nm** | **Experimental emission wavelength / nm** |
| --- | --- | --- | --- | --- | --- |
| **Au-DMAP** | S_1_ | H → L (99.4 %) | 2.7162 | 456.47 | 460 |
| **Au-DMAP** | S_2_ | H-1 → L (99.3%) | 2.8023 | 442.44 |  |
| **Au-DMAP** | S_3_ | H-2 → L (99.5%) | 3.7159 | 333.66 |  |
| **Au-DMAP** | S_4_ | H → L+1 (98.9%) | 3.9623 | 312.91 |  |
| **Au-DMAP** | S_5_ | H-1 →L+1 (98.7%) | 4.0468 | 306.38 |  |
| **Mn-DMAP** | S_1_ | H → L (97.3%) | 2.3175 | 534.99 | 530 |
| **Mn-DMAP** | S_2_ | H → L+1 (96.8%) | 2.5075 | 494.45 |  |
| **Mn-DMAP** | S_3_ | H-1 → L (95.3%) | 2.7964 | 443.36 |  |
| **Mn-DMAP** | S_4_ | H-1 → L+1 (75.4%) | 6.4961 | 190.86 |  |
| **Mn-DMAP** | S_5_ | H-2 → L (70.1%) | 7.0382 | 176.16 |  |
| **In-DMAP** | S_1_ | H → L (99.9%) | 2.0863 | 594.29 | 580 |
| **In-DMAP** | S_2_ | H-1 → L (99.9%) | 2.4284 | 510.56 |  |
| **In-DMAP** | S_3_ | H → L+1 (99.9%) | 3.1336 | 395.66 |  |
| **In-DMAP** | S_4_ | H-2 → L (99.8%) | 3.4005 | 364.61 |  |
| **In-DMAP** | S_5_ | H-1 → L+1 (99.9%) | 3.4758 | 356.70 |  |

Table S4. The comparison of the wavelength and OLC of other optical waveguides materials and M-DMAP under ambient conditions.

| **Name** | **Wavelength/ nm** | **OLC/ dB mm^-1^** |
| --- | --- | --- |
| Au-DMAP (this work) | 460 | 2.84 |
| In-DMAP (this work) | 580 | 3.22 |
| Mn-DMAP (this work) | 530 | 7.93 (short axis) / 9.53 (long axis) |
| PDI [84] | 688 | 130 |
| DPEpe-HCl [85] | 630 | 18.1 |
| BCZ [86] | 567 | 33 |
| DCF [87] | 560 | 23.9 |
| Eu-BTC [88] | 615 | 12 |
| Tb-BTC [88] | 543 | 25 |
| Eu dopped Tb-BTC [88] | 615 | 33 |
| FTCs [89] | 550 | 18.1 |
| (KC)MnCl_4_ [90] | 518 | 9.93 |
| OIHP-AD [91] | 533 | 4 |
| Pt_1_Ag_18_[25] | 600 | 5.26 |
| Au_x_Ag_19–x_ (7 ≤ x ≤ 9)[25] | 770 | 7.77 |

Table S5. PL lifetimes constants and the pre-exponential coefficients of organic metal halides.

| **Sample** | ***λ*_ex_ (nm)** | ***λ*_em_ (nm)** | ***τ*_i_** | **A_i_ (%)** | **<τ>** | ***χ*^2^** |
| --- | --- | --- | --- | --- | --- | --- |
| Au-DMAP | 280 | 462 | 1.42 ns | 33.13 | 1.15 ns | 1.031 |
|  |  |  | 1.02 ns | 66.87 |  |  |
| In-DMAP | 280 | 530 | 1.18 ns | 35.72 | 1.53 ns | 1.011 |
|  |  |  | 1.73 ns | 64.28 |  |  |
| Mn-DMAP | 280 | 582 | 5.11 ns | 27.28 | 4.07 ns | 1.087 |
|  |  |  | 3.68 ns | 72.72 |  |  |

*τ*_i_ is the excited state lifetime. A_i_ represents the ratio of *τ*_i_. The fitting goodness is manifested by the value of χ^2^. In the double-exponential case, <*τ*> = A_1_*τ*_1_ + A_2_*τ*_2_, A_1_ + A_2_ = 1.
