# Supplementary figures and images for: Flexible Crystal Heterojunctions of Low-Dimensional Organic Metal Halides Enabling Color-Tunable Space-Resolved Optical Waveguides
Source: Research (Wash D C). 2023 Oct 30;6:0259. doi: 10.34133/research.0259 (PMC10616971; doi:10.34133/research.0259)

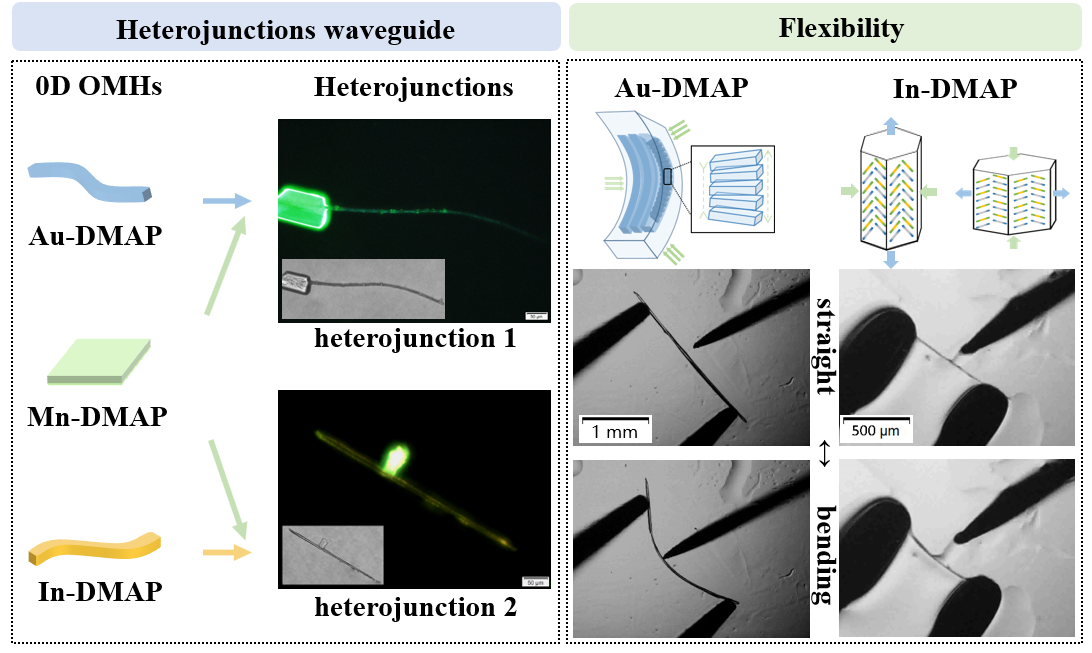

Supplement: Supplementary 1 — Figs. S1 to S9 Tables S1 to S5 References [84–89] [file research.0259.f1.zip › TOC.png]
